# Inactivation of hypocretin receptor-2 signaling in dopaminergic neurons induces hyperarousal and enhanced cognition but impaired inhibitory control

**DOI:** 10.1038/s41380-023-02329-z

**Published:** 2023-12-21

**Authors:** Mojtaba Bandarabadi, Sha Li, Lea Aeschlimann, Giulia Colombo, Stamatina Tzanoulinou, Mehdi Tafti, Andrea Becchetti, Benjamin Boutrel, Anne Vassalli

**Affiliations:** 1 Department of Biomedical Sciences, University of Lausanne, Lausanne, Switzerland; 2 Centre for Psychiatric Neuroscience, Department of Psychiatry, The Lausanne University Hospital, Lausanne, Switzerland; 3 Department of Biotechnology and Biosciences, University of Milano-Bicocca, Milano, Italy

**Keywords:** Neuroscience, Biomarkers

## Abstract

Hypocretin/Orexin (HCRT/OX) and dopamine (DA) are both key effectors of salience processing, reward and stress-related behaviors and motivational states, yet their respective roles and interactions are poorly delineated. We inactivated HCRT-to-DA connectivity by genetic disruption of *Hypocretin receptor-1* (*Hcrtr1*), *Hypocretin receptor-2* (*Hcrtr2*), or both receptors (*Hcrtr1&2*) in DA neurons and analyzed the consequences on vigilance states, brain oscillations and cognitive performance in freely behaving mice. Unexpectedly, loss of *Hcrtr2*, but not *Hcrtr1* or *Hcrtr1&2*, induced a dramatic increase in theta (7–11 Hz) electroencephalographic (EEG) activity in both wakefulness and rapid-eye-movement sleep (REMS). DA^*Hcrtr2*^*-*deficient mice spent more time in an active (or theta activity-enriched) substate of wakefulness, and exhibited prolonged REMS. Additionally, both wake and REMS displayed enhanced theta-gamma phase-amplitude coupling. The baseline waking EEG of DA^*Hcrtr2*^*-*deficient mice exhibited diminished infra-theta, but increased theta power, two hallmarks of EEG hyperarousal, that were however uncoupled from locomotor activity. Upon exposure to novel, either rewarding or stress-inducing environments, DA^*Hcrtr2*^*-*deficient mice featured more pronounced waking theta and fast-gamma (52–80 Hz) EEG activity surges compared to littermate controls, further suggesting increased alertness. Cognitive performance was evaluated in an operant conditioning paradigm, which revealed that DA^*Hcrtr2*^-ablated mice manifest faster task acquisition and higher choice accuracy under increasingly demanding task contingencies. However, the mice concurrently displayed maladaptive patterns of reward-seeking, with behavioral indices of enhanced impulsivity and compulsivity. None of the EEG changes observed in DA^*Hcrtr2*^*-*deficient mice were seen in DA^*Hcrtr1*^*-*ablated mice, which tended to show opposite EEG phenotypes. Our findings establish a clear genetically-defined link between monosynaptic HCRT-to-DA neurotransmission and theta oscillations, with a differential and novel role of HCRTR2 in theta-gamma cross-frequency coupling, attentional processes, and executive functions, relevant to disorders including narcolepsy, attention-deficit/hyperactivity disorder, and Parkinson’s disease.

## Introduction

Neuromodulators are master levers of brain circuits which shape brain states and functional output by tuning neuronal firing or synaptic strength. Hypocretin (HCRT, also known as Orexin, OX) and dopamine (DA) are both major neuromodulators of arousal and motivated states. Their interactions, however, remain ill defined. A small population of glutamatergic neurons in the lateral, perifornical and dorsomedial hypothalamus synthesizes the neuropeptides HCRT-1 and HCRT-2 (OXA and OXB), and sends axonal projections to all wake-promoting monoaminergic (including dopaminergic) and cholinergic nuclei of the ascending arousal system, as well as directly innervates their targets, the neocortex, thalamus, hippocampus, amygdala, and spinal cord [1]. HCRT neurons thus establish a brain-wide neural network, with extraordinarily pleiotropic functions, spanning multiple physiological, behavioral, emotional, and temporal domains [2, 3]. HCRT peptides act through two genetically independent and differentially expressed G-protein-coupled-receptors, HCRTR1 and HCRTR2. HCRTR2 binds both peptides, whereas HCRTR1 only binds HCRT-1 with high affinity. Brain level of each peptide, differential signaling via the two receptors, and how each uniquely impacts vigilance states and behavior remain elusive.

HCRT neurons fire maximally during active wakefulness, in line with their role in maintaining heightened arousal [4], but can also show burst firing during phasic events of rapid-eye-movement sleep (REMS) [4], and occasional bursting during non-REMS (NREMS) [5]¸ consistent with their role in sleep-to-wake transitions [6]. An unexpected role of HCRT neurons in REMS was recently discovered [7]. Disrupting the HCRT system in mice [8], dogs [9], and humans [10] causes narcolepsy-type-1, a disease characterized by excessive daytime sleepiness, vigilance state fragmentation, hypnagogic/hypnopompic hallucinations, and emotionally-driven sudden muscle atonia, or cataplexy [11]. Inactivation of the *Hcrt* gene, or combined loss of the two receptors, are sufficient to induce narcolepsy in mice [12, 13], indicating that narcolepsy stems from deficient HCRTR signaling. However, a circuit-based understanding of the unique role of each HCRTR1 or HCRTR2-expressing target population is still lacking. Among HCRT targets, DA neurons are particularly interesting because of their established prime role in regulating arousal and arousal-dependent behaviors [14] and apparent functional overlap with HCRT.

Daytime sleepiness and cataplexy are two main narcolepsy symptoms with opposite manifestations, EEG hypoarousal and hyperarousal, respectively, yet both stem from HCRT deficiency and both respond to dopaminergic drugs. In narcoleptic dogs and mice, sleep attacks respond to D1-receptor agonists, and cataplexy responds to D2/D3-receptor antagonists [15]. Most stimulants act by raising brain DA levels. These findings suggested that DA is an important effector of the HCRT system, and indeed HCRT neurons densely innervate the VTA dopaminergic (DA^VTA^) system [16]. HCRT-1 dose-dependently elevates [Ca^2+^]_intracell_ in dissociated DA^VTA^ cells [17], and both HCRT peptides enhance DA^VTA^ firing and induce tetrodotoxin-resistant depolarization in brain slices [18]. Different firing patterns were elicited in different DA^VTA^ cell subsets, and single cells expressed either *Hcrtr1*, *Hcrtr2*, or both receptors [18]. Whether the different responses reflected differential *Hcrtr1* vs *Hcrtr2* expression remains an open question.

HCRT is thought to stimulate DA^VTA^ activity both by direct HCRTR activation on DA somatodendritic compartment, and indirectly by potentiating glutamatergic afferents [18–20]. Intra-VTA HCRTR1 antagonism precluded acquisition of cocaine-induced locomotor sensitization in rats, and blocked cocaine-induced DA^VTA^ glutamatergic potentiation ex vivo [19], suggesting that HCRT → DA^VTA^ pathways are implicated in drug-associated plasticity. Because HCRT neurons sense reward and danger-predicting cues [21], and DA^VTA^ glutamatergic potentiation is a mechanism of value-driven learning of salient stimuli, the HCRT → DA^VTA^ circuit is positioned to be a prime inducer of DA^VTA^ potentiation-related positive and negative reinforcement [22]. For instance, male copulatory behavior is coupled to FOS activation in HCRT terminal-apposed DA^VTA^ cells, and HCRTR1 antagonism suppresses mating behavior [23]. HCRT-induced DA^VTA^ activation is thus linked to rewards, but also to stress-associated arousal [24]. In rat models of stress-induced psychosis-like behavior associated with DA^VTA^ hyperactivity, HCRTR1/R2-dual antagonism reversed both aberrant DA^VTA^ activity and behavioral correlates of psychosis [25]. Intracerebroventricular HCRT-1 infusion induced hyperlocomotion, stereotypy and grooming, which could be fully abolished by DA-receptor antagonism [17]. Studies therefore implicate HCRT → DA^VTA^ circuits in drug-associated plasticity, sexual approach, stress-induced chronic arousal (e.g. post-traumatic stress) and several other neuropsychiatric disorders [26].

The role of DA^VTA^ neurons in sleep/wake control was long ignored because average cell spiking rate changes little across vigilance states. However spiking patterns vary sharply, with prominent DA^VTA^ burst firing in active wakefulness and REMS, but tonic activity during NREMS [27]. The causal role of DA^VTA^ activation in salience-induced arousal is now demonstrated [28, 29]. Interestingly, a major VTA target is the nucleus accumbens (NAc), and the DA^VTA^ → NAc circuit, well-known to mediate reward-driven motivated behavior, recently emerged as critical in bridging motivation and vigilance state control [29, 30]. Hence motivation was suggested as ‘3^rd^ process’ of sleep/wake regulation [31, 32]. Altogether, HCRT and DA neurons, on their own, and through HCRT → DA^VTA^ pathways, are implicated in emotional drive, salience processing, behavioral state transitions and cognition, but delineation of their interplay, and how it may be implicated in arousal disorders, remain underexplored. In addition to the DA^VTA^ cell group, other DA cell nuclei, notably in the hypothalamus and dorsal raphe, are known to be implicated in vigilance state regulation [33–35]. Whether they are targets of HCRT neurons and express HCRT receptors is however unknown.

To functionally interrogate HCRT → DA circuits, we generated mice whose dopaminergic system cannot respond to HCRT input, via HCRTR1, or HCRTR2, or neither. Importantly, these genetically-targeted mice interrogate all types of HCRT → DA connectivity, whether endowed by post-, pre-, or extra-synaptic action of HCRT binding receptors on DA cell somata, dendrites or axons [36, 37], i.e., in dopaminergic nuclei or their targets. The mice also functionally interrogate all potential HCRT → DA neuronal group connectivity, not only HCRT → DA^VTA^. Our findings place DA signaling under prominent neuromodulatory control of the hypocretinergic system and show that this neuromodulation operates differentially through HCRTR1 and HCRTR2. We reveal prominent roles of HCRTR2-mediated HCRT → DA neurotransmission in regulating brain oscillations, learning, attention, and behavioral inhibition, with clinical implications for HCRTR-targeted drug development.

## Results

### Selective inactivation of HCRT receptors in dopaminergic neurons

**Fig. 1:**
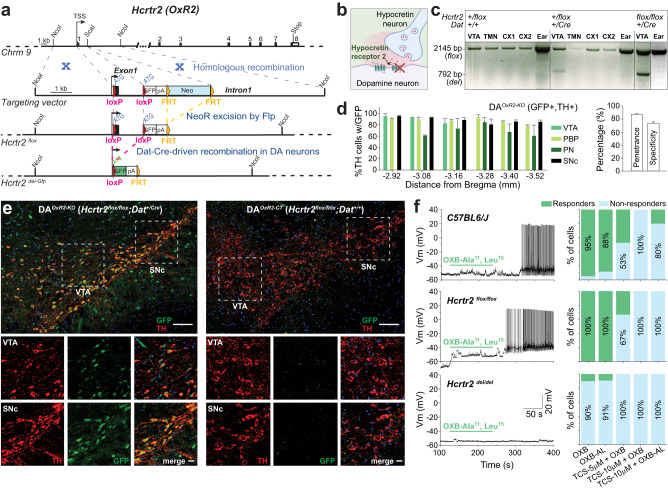
Generation of mice with selective disruption of *Hcrtr2* in dopamine neurons. **a** Homologous recombination of the *Hcrtr2* gene with the targeting vector creates the *Hcrtr2-flox* allele. The 5’loxP site was inserted in Exon1 5’-untranslated-region. 3’loxP was inserted within Intron1. In *Dat-IRES-Cre*-expressing neurons, CRE/lox recombination creates the *Hcrtr2*^*del-GFP*^ allele, with genomic deletion of DNA encoding 74 aa, encompassing HCRTR2 signal peptide, N-terminal domain, and most transmembrane region 1, and replacement of the *Hcrtr2* coding sequence with a promoterless *Gfp* cassette. The endogenous *Hcrtr2* promoter now drives *Gfp* instead of *Hcrtr2* in DA neurons, marking cells having lost *Hcrtr2* expression with GFP. TSS, Transcription start site. pA, polyadenylation signal. Chrm9, Chromosome 9. *Hcrtr2*^*flox*^ is *Hcrtr2*^*tm1.1Ava*^ (MGI:5637402), and *Hcrtr2*^*KO-Gfp*^ is *Hcrtr2*^*tm1.2Ava*^ (MGI: 5637403). **b** Schematic representation of *DA*^*OxR2-KO*^ mice. **c** Evidence of tissue-specific genomic recombination. DNA was isolated from various tissues and subject to PCR. Unrecombined *HcrtR2*-flox diagnostic band (Flox, 2,145 bp) is observed in cortex, TMN, VTA and ear from DA^*OxR2-CT*^ and DA^*OxR2-KO*^ mice, while the knockout diagnostic band (KO; 792 bp) is only observed in VTA of DA^*OxR2-KO*^ mice. The 792 bp recombined fragment was fully sequenced, confirming exact recombination event (*n* = 2). **d** GFP and Tyrosine Hydroxylase (TH) immunostaining demonstrates efficiency and specificity of *Hcrtr2* Exon1 deletion in DA cells of the ventral midbrain (−2.92 to −3.88 mm from bregma) of *DA*^*OxR2-KO*^ mice. (Left) Quantification in several midbrain subregions. (Right) Overall penetrance (% of TH^+^ neurons co-expressing GFP) was 87.2 ± 1.5% (*n* = 12 sections, 2 mice). Specificity (% of GFP^+^ cells co-expressing TH) was 73.2 ± 2.4% (*n* = 12 sections, 2 mice). VTA, ventral tegmental area; PBP, parabrachial pigmented nucleus; PN paranigral nucleus, SNc substantia nigra pars compacta. **e** Representative confocal images depicting TH and GFP co-localization in ventral midbrain of DA^*OxR2-KO*^, but not DA^*OxR2-CT*^, mice. Coronal 20-µm brain sections at −3.08 mm from bregma. Scale bar: low magnification, 100 µm; high magnification, 20 µm. **f** Electrophysiological demonstration that *Hcrtr2* Cre/lox recombination inactivates HCRTR2. (Left) Voltage trace recordings from putative histaminergic neurons in TMN of *C57BL/6* *J*, *Hcrtr2*^*flox/flox*^, and *Hcrtr2*^*del/del*^ mice. Cells were held at −50 mV in current-clamp mode. Voltage traces represent 5-min continuous recordings, before, during (green horizontal line), and after OXB-AL (200 nM) application. OXB-AL triggers a long train of action potentials in neurons from *C57BL/6J* and *Hcrtr2*^*flox/flox*^, but not *Hcrtr2*^*del/del*^, mice. (Right) Percentage of neurons responding to different treatments in each genotype. Treatments were as follows: OXB (100 nM); OXB-AL (200 nM); TCS (5 μM) + OXB (100 nM); TCS (10 μM) + OXB (100 nM); TCS (10 μM) + OXB-AL (200 nM). Number of neurons per treatment: *C57BL/6J*: 19/17/17/8/5; *Hcrtr2*^*flox/flox*^: 4/3/6/5/6; *Hcrtr2*^*del/del*^: 10/11/7/5/5). OXB-AL: [Ala^11^, D-Leu^15^]-Orexin B; TCS: TCS-OX2-029; TMN: tuberomammillary nucleus.

To conditionally inactivate HCRT receptors, we engineered the *Hcrtr1* (*OxR1*) and *Hcrtr2* (*OxR2*) genes and created Cre-dependent knockout/GFP-reporter floxed alleles: *Hcrtr1*^*flox*^ (reported in [38]), and *Hcrtr2*^*flox*^ (Fig. 1). Each floxed line was independently crossed to a *Dopamine transporter* Cre driver (*Dat-IRES-Cre* [39]), generating *Hcrtr1*^*flox/flox*^*;Dat*^*+/Cre*^ (abbreviated: DA^*OxR1-KO*^), *Hcrtr2*^*flox/flox*^*;Dat*^*+/Cre*^ (DA^*OxR2-KO*^), and compound *Hcrtr1*^*flox/flox*^*;Hcrtr2*^*flox/flox*^*;Dat*^*+/Cre*^ (DA^*OxR1&2-KO*^) mutant mice (Fig. 1 and Fig. S1). Because floxed genetic alleles can show altered expression relative to *wild-type* we established crosses that generate *Hcrtr1*^*flox/flox*^, or *Hcrtr2*^*flox/flox*^ littermates, to use as genetic control group (CT) for each KO group: *Hcrtr1*^*flox/flox*^ (DA^*OxR1-CT*^), *Hcrtr2*^*flox/flox*^ (DA^*OxR2-CT*^), and *Hcrtr1*^*flox/flox*^*;Hcrtr2*^*flox/flox*^ (DA^*OxR1&2-CT*^). We therefore generated 6 genotypic groups (3 KO:CT pairs, for *Hcrtr1*, *Hcrtr2*, and *Hcrtr1&2*, respectively), and performed all analyses as pair-wise comparisons between KO and CT littermate groups.

To verify at nucleotide level the accuracy and DA-specificity of Cre/lox *Hcrtr2* gene recombination in vivo, we sequenced genomic DNA of various tissues from DA^*OxR2-KO*^ and DA^*OxR2-CT*^ mice. Only VTA of DA^*OxR2-KO*^ mice contained the diagnostic 792 bp recombinant fragment, absent in tuberomammillary nucleus (TMN), neocortex, or ear (Fig. 1c). Because not all DA neurons express *Dat* [40], we estimated the fraction of DA neurons expressing *Dat-ires-Cre* by counting cells immunoreactive for CRE and tyrosine hydroxylase (TH), and found that overall 88.4% of ventral midbrain area TH^+^ neurons express *Dat-ires-Cre* (2640 TH^+^ cells, n = 4 mice; Fig. S2), and are therefore susceptible to Cre/lox recombination.

We quantified efficiency of *Dat*-Cre-driven gene deletion in DA neurons using our designed GFP-reporter of gene inactivation, and found that 83.0 ± 2.8%, and 87.2 ± 1.5%, of ventral midbrain TH^+^ neurons co-expressed GFP in DA^*OxR1-KO*^, and DA^*OxR*2-KO^ mice, respectively (Fig. S1d, e for R1, Fig. 1d, e for R2; *n* = 12 sections, 2 mice per group), and have thus successively recombined. These fractions represent the % of TH^+^ cells that underwent recombination and hold an active endogenous *Hcrtr1* or *Hcrtr2* promoter driving the GFP-reporter. This demonstrates that a majority of ventral midbrain DA neurons express *Hcrtr1*, or *Hcrtr2* (>83% and >87%, respectively). Anti-HCRTR1 immunostaining confirmed that most TH^+^ DA^VTA^ cells lost HCRTR1-immunoreactivity in DA^*OxR*1-KO^, but not DA^*OxR*1-CT^, mice (Fig. S1f), and now express GFP instead (Fig. S1e). DA-specificity of *Hcrtr1 and Hcrtr2* gene deletion was quantified by counting the fraction of GFP^+^ neurons expressing TH, confirming that a majority of recombined GFP^+^ cells are indeed DA neurons (DA^*OxR*1-KO^: 74.4 ± 0.5%, Fig. S1d; DA^*OxR*2-KO^: 73.19 ± 2.42%, Fig. 1d; *n* = 12 sections, 2 mice per group).

We next sought to functionally assess the effect of *Hcrtr2* Cre/lox recombination and verify that recombination creates a null (*Hcrtr2*^*del*^), while the unrecombined *Hcrtr2*^*flox*^ gene remains functional in non-DA cells. Thus we performed patch clamp recordings in ventral TMN histamine neurons, a cell type expressing *Hcrtr2* and not appreciably *Hcrtr1* [41], using brain slices of *wild-type* (*C57BL/6J), Hcrtr2*^*flox/flox*^, and whole-body KO (*Hcrtr2*^*del/del*^) mice (see Methods). Putative histamine neurons were identified using their electrophysiological characteristics with post-recording morphological confirmation of biocytin-loaded cells (Fig. S3, Table S1, see Supplementary Methods). We found that application of the selective HCRTR2-agonist [Ala^11^,D-Leu^15^]-Orexin B (OXB-AL) triggers spike trains in histamine neurons of *C57BL/6J* (88% of cells), and *Hcrtr2*^*flox/flox*^ mice (100% of cells), but elicits little response in cells from *Hcrtr2*^*del/del*^ mice (9% of cells) (Fig. 1f). Application of the selective HCRTR2-antagonist TCS-OX2-29 fully blocked the effects of OXB and OXB-AL in >80% cells of *C57BL/6J* and *Hcrtr2*^*flox/flox*^ mice, confirming HCRTR2-dependency of the response. Therefore, creation of the *Hcrtr2*^*flox*^ allele has preserved its function, while the post-recombination *Hcrtr2*^*del*^ allele is inactive.

### Inactivating *Hcrtr2* in DA neurons causes spontaneous electrocortical hyperarousal

**Fig. 2:**
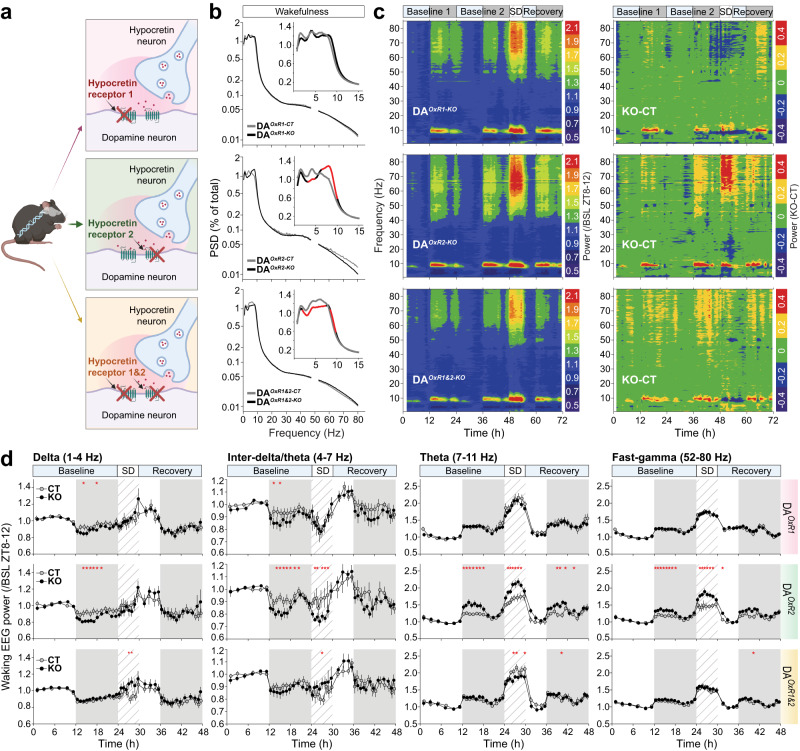
Dopaminergic *Hcrtr2*-ablated mice exhibit theta and fast-gamma enriched wakefulness. **a** Schematic representation of DA^*OxR1-KO*^, DA^*OxR2-KO*^, and DA^*OxR1&2-KO*^ mice. **b** EEG power spectral density (PSD) analysis of wakefulness in the 3 mutant and control groups averaged across 2 baseline days. PSD values are expressed as percentage of a baseline total power density reference value calculated for each mouse (see SI Methods). Red lines indicate frequency ranges with significant differences. Note logarithmic Y-scales. Insets magnify EEG spectra across 0.75–15 Hz with linear Y-axis. DA^*OxR2-KO*^ mice show lower delta power (across 3.25–4.75 Hz), but higher theta power (across 6.75–9.75 Hz) compared to DA^*OxR2-CT*^ mice (two-way ANOVA; genotypeXfrequency interaction F(280,4502) = 1.858, *P* < 0.001; Tukey post-hoc test, *P* < 0.05). Wakefulness of DA^*OxR1&2-KO*^ mice also display lower delta (2–4 Hz), but diminished power extends through inter-delta/theta and slow-theta frequencies (4–7.25 Hz). Only a narrow fast-theta band (8.5–9.75 Hz) shows increased power (two-way ANOVA; genotypeXfrequency interaction F(286,4879) = 1.175, *P* = 0.026; Tukey post-hoc test, *P* < 0.05). **c** Time-frequency heatmaps on the left depict dynamics of the waking EEG in the 3 KO lines across the 3-day recordings as schematized on top. Color-coding represents EEG power calculated across time for each 0.25-Hz frequency bin and expressed relative to mean baseline (BSL) wakefulness during the light phase last 4 h (ZT8-12). Heatmaps on the right depict differential power between KO and controls (CT values are subtracted from KO values, KO-CT). **d** Time-course analysis of waking delta (1–4 Hz), inter-delta/theta (4–7 Hz), theta (7–11 Hz), and fast-gamma (52.5–80 Hz) band powers. Depicted are EEG powers during wakefulness across time in (averaged) 2 baseline days, 6-h SD and 18 h of recovery, expressed relative to their mean values in baseline ZT8-12 wakefulness (two-way ANOVA; Delta: DA^*OxR2-KO*^: baseline: genotype effect F(1,17) = 39.615, *P* < 0.001; Inter-delta/theta: DA^*OxR2-KO*^: baseline: genotype effect F(1,17) = 20.667, *P* < 0.001; SD: genotype effect F(1,23) = 17.107, *P* < 0.001; Theta: DA^*OxR2-KO*^: baseline: genotype effect F(1,17) = 33.896, *P* < 0.001, genotypeXtime interaction F(17,288) = 2.159, *P* = 0.005; SD: genotype effect F(1,23) = 84.493, *P* < 0.001, genotypeXtime interaction F(23,384) = 1.896, *P* = 0.008; DA^*OxR1&2-KO*^: SD: genotype effect F(1,23) = 10.676, *P* < 0.001; Fast-gamma: DA^*OxR2-KO*^: baseline: genotype effect F(1,17) = 31.217, *P* < 0.001, genotypeXtime interaction F(17,288) = 1.693, *P* = 0.043; SD: genotype effect F(1,23) = 73.561, *P* < 0.001, genotypeXtime interaction F(23,384) = 2.085, *P* = 0.003; DA^*OxR1&2-KO*^: baseline: genotype effect F(1,17) = 6.551, *P* = 0.011; SD: genotype effect F(1,23) = 5.922, *P* = 0.015; Bonferroni post-hoc test, *P < 0.05). *n* = 9 mice per group, except *n* = 10 for DA^*OxR1&2-CT*^.

To determine how disrupting HCRT → DA neurotransmission affects vigilance states and brain oscillations, we performed EEG/EMG recordings in freely-behaving mice. While the quantity of wakefulness and NREMS did not show major differences between KO and CT mice in any group (Fig. S4), wakefulness spectral quality showed profound alterations. DA^*OxR2-KO*^ mice featured markedly higher waking theta (7-11 Hz) power, but lower delta (1–4 Hz) power, compared to DA^*OxR2-CT*^ mice (see exact affected frequency ranges in Fig. 2b and legend). Because waking theta activity is associated with exploration and arousal [42], whereas waking delta and inter-delta/theta (4–7 Hz) (together referred to as ‘infra-theta’, 1–7 Hz) are markers of sleep propensity and decreased vigilance [43, 44], these EEG alterations suggest that DA^*OxR2-KO*^ mice may be spontaneously more alert than controls. In contrast, DA^*OxR1-KO*^ mice’ waking spectra did not differ from controls, and wakefulness of DA^*OxR1&2-KO*^ mice featured diminished power across a wide infra-theta range (2.00–7.25 Hz; Fig. 2b).

After 2 days of baseline recording, at light-onset of Day-3 (i.e, in early resting phase), we exposed mice to 6-hour of ‘gentle handling’ sleep deprivation (SD), followed by 18 h of recovery. Time-frequency heatmap analysis of the waking EEG during these 3 days revealed powerful surges in theta and fast-gamma (52-80 Hz) power above baseline at times of elevated activity (dark phase and SD) in all genotypes, as expected for periods of increased alertness [45], but these effects were most pronounced in DA^*OxR2-KO*^ mice (Fig. 2c, left heatmap, middle). When EEG power differences between KO and CT were extracted by subtracting CT values from KO values (KO-CT), it became apparent that DA^*OxR2-KO*^ mice theta and fast-gamma power surges surpassed those of CT mice (Fig. 2c, right heatmap, middle). As theta and fast-gamma increased, infra-theta and beta (15-30 Hz) frequencies concomitantly decreased, most prominently during SD. In striking contrast, SD induced a drop in theta power in DA^*OxR1&2-KO*^ mice relative to controls (Fig. 2c, right heatmap, bottom), suggesting that dual *Hcrtr1&2 vs* single *Hcrtr2* loss in DA neurons cause opposite responses in alertness at times of high sleep pressure and experimental handling. To further address quantitatively these spectral trends, we analyzed the waking EEG power dynamics within specific frequency ranges of interest. This confirmed that theta and fast-gamma EEG activities dramatically increase in DA^*OxR2-KO*^ mice relative to controls during dark periods and SD, while delta and inter-delta/theta activities concomitantly decline (Fig. 2d). DA^*OxR1&2-KO*^ mice exhibit the opposite, with decreased theta, but higher delta and inter-delta/theta during SD (Fig. 2d), and interestingly increased beta (15–30 Hz) activity as well (Fig. S6). Thus, as estimated by both vigilance (theta) and sleepiness (infra-theta) EEG indices, our results suggest that dopaminergic *Hcrtr2* disruption increases alertness, whereas combined *Hcrtr1&2* loss reduces it. Beta band enhancement in DA^*OxR1&2-KO*^ animals in periods of heightened locomotor activity (SD), is intriguing and reminiscent of observations in DA-depleted rats and in Parkinson’s disease (PD)-associated movement disorders [46, 47].

To address how disrupting HCRT → DA signaling affects wakefulness in challenging environments, we exposed animals to either enriched or stress-inducing environments **(**Fig. S7, see Methods). Exposure to nesting material, which is rewarding in rodents, was associated with surges in theta and fast-gamma activity in all genotype groups, however these surges appeared stronger and longer-lasting in DA^*OxR1-KO*^ and DA^*OxR2-KO*^ mice, compared to controls (Fig. S7b). Strikingly, again, in double DA^*OxR1&2-KO*^ mutants, nest material induced an opposite EEG response, with a decline in theta and fast-gamma activity in the 3 h following Nestlet addition (Fig. S7b). We next tested the mice’ EEG response upon removal from the nest at time of high sleep propensity (ZT3), and transfer to a foreign environment. This manipulation induced theta and fast-gamma power increase in DA^*OxR2-KO*^, but decrease in DA^*OxR1&2-KO*^ mice, relative to controls (Fig. S7c, d). Thus, the spectral quality of wakefulness consistently shows opposite changes in DA^*OxR2-KO*^ and DA^*OxR1&2-KO*^ mice, in both rewarding and stressful environments, suggesting that HCRT → DA circuits play prominent roles in regulating both spontaneous and stimuli-induced arousal, irrespective of whether stimuli have positive or negative valence.

### DA^*OxR2-KO*^ mice display increased theta-dominated wakefulness uncoupled from locomotion

**Fig. 3:**
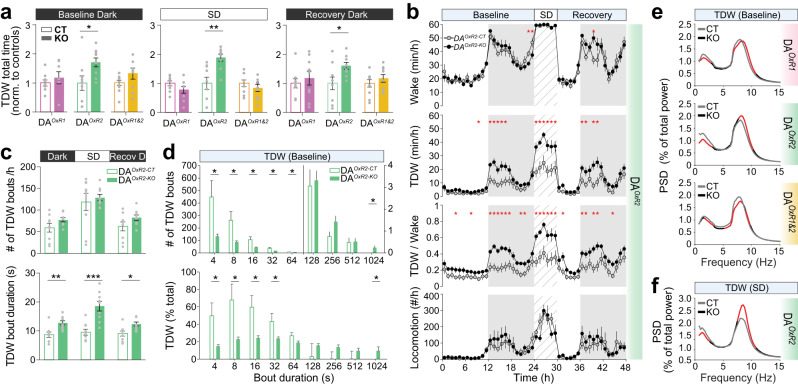
Dopaminergic *Hcrtr2*-ablated mice upregulate a theta-dominated waking state uncoupled from locomotion. **a** Total time spent in theta-dominated wakefulness (TDW) in DA^*OxR1-KO*^, DA^*OxR2-KO*^, and DA^*OxR1&2-KO*^ mice, normalized to their controls. DA^*OxR2-KO*^ mice show profound increases in TDW time during dark and SD periods (baseline dark: *P* = 0.027; SD: *P* = 0.002 recovery dark: *P* = 0.028; independent *t*-test). **b** Time-courses of wakefulness, TDW, TDW-to-wakefulness ratio (TDW/W), and locomotor activity in DA^*OxR2-KO*^ and DA^*OxR2-CT*^ mice. Wakefulness of DA^*OxR2-KO*^ mice is profoundly enriched in a theta-dominated state uncoupled from locomotion, during dark and SD periods (two-way ANOVA, genotype effect; baseline TDW: F(1,23) = 52.804, *P* < 0.001; SD TDW: F(1,23) = 97.222, *P* < 0.001; baseline TDW/W: F(1,23) = 127.572, *P* < 0.001; SD TDW/W: F(1,23) = 133.609, *P* < 0.001; Bonferroni post-hoc test, *P < 0.05). Locomotor activity was unchanged between DA^*OxR2-KO*^ mice and controls (two-way ANOVA, genotype effect; baseline: F(1,23) = 0.863, *P* = 0.354; SD: F(1,23) = 0.110, *P* = 0.740). **c** Number and duration of TDW bouts in DA^*OxR2-KO*^ and DA^*OxR2-CT*^ mice. Mean duration of TDW bouts was increased in DA^*OxR2-KO*^ mice during baseline dark, SD, and recovery dark periods (DA^*OxR2-KO*^ vs DA^*OxR2-CT*^; baseline dark: *P* = 0.0028; SD: *P* < 0.001; recovery dark: *P* = 0.0083; independent *t*-test). The number of TDW bouts however did not differ between genotypes. **d** TDW bout duration distributions during 48 h baseline reveals that DA^*OxR2-KO*^ mice display less (Top) and a smaller fraction of total TDW time (Bottom) in bouts lasting ≤64 s, but more and a larger fraction of total TDW in >1024s-long bouts (two-way ANOVA; distribution of bout number: genotypeXduration interaction F(8,144) = 4.646, *P* < 0.001; distribution of relative time: genotypeXduration interaction F(8,144) = 4.049, *P* < 0.001; Bonferroni post-hoc test, *P < 0.05). Only the lower bin limit of bout durations is indicated for 4, 8–12, 16–28, 32–60, 64–124, 128–252, 256–508, 512-1020, >1024 s long bouts. **e** Baseline TDW spectra (averaged across 2 days). Power spectral density (PSD) values expressed as in Fig. 2b. Red lines indicate frequency ranges with significant differences. DA^*OxR1-KO*^ mice show lower delta (1-2.75 Hz) and slow-theta (6.75-8 Hz), but higher fast-theta (8.5–10.75 Hz) powers compared to controls (two-way ANOVA; genotypeXfrequency interaction F(288,4624) = 1.419, *P* < 0.001; Tukey post-hoc test, *P* < 0.05). DA^*OxR1&2-KO*^ mice show similar spectra with a more pronounced decrease in slow-theta (two-way ANOVA; genotypeXfrequency interaction F(286,4879) = 1.302, *P* < 0.001; Tukey post-hoc test, *P* < 0.05). In contrast, DA^*OxR2-KO*^ mice show increased theta power density across 7.75-10.25 Hz. (two-way ANOVA; genotypeXfrequency interaction F(280,4496) = 1505, *P* < 0.001; Tukey post-hoc test, *P* < 0.05). **f** TDW spectra during SD reveal an even further increase in theta power in DA^*OxR2-KO*^ compared to DA^*OxR2-CT*^ mice (two-way ANOVA; genotypeXfrequency interaction F(280,4496) = 3.514, *P* < 0.001; Tukey post-hoc test, *P* < 0.05). Bar graphs depict mean ± SEM. n = 9 mice per group, except *n* = 10 for DA^*OxR1&2-CT*^.

Prominent theta activity is present during locomotion and exploratory behavior in rodents, but also during alert immobility states [48]. We next examined whether increased waking theta power of DA^*OxR2-KO*^ mice results from altered sub-states of wakefulness. We first quantified theta-dominated wakefulness (TDW) [45] in all 6 groups and found that although total time awake is not majorly affected in any group (Fig. S4a, b), time spent in TDW markedly increased in DA^*OxR2-KO*^, but not DA^*OxR1-KO*^ or DA^*OxR1&2-KO*^, mice during baseline dark, SD, and recovery dark periods (DA^*OxR2-KO*^ vs DA^*OxR2-CT*^: baseline dark: 196.3 ± 17.7 vs 114.9 ± 28.5 min, SD: 239.2 ± 15.4 vs 126.7 ± 26.7 min, recovery dark: 202.1 ± 14.6 vs 125.9 ± 27.8 min, Fig. 3a). Time-course analyses reveal that TDW time (min/h), and fraction of wakefulness occupied by TDW (TDW/W ratio) are dramatically increased in DA^*OxR2-KO*^ in the first halves of the night and during SD (Fig. 3b), while total wakefulness is unchanged (Fig. 3b, top). We next assessed whether the increased TDW correlates with locomotion and found no alteration in locomotor activity in DA^*OxR2-KO*^ mice compared to controls (Fig. 3b, bottom, Fig. S8), suggesting that DA^*OxR2-KO*^ mice display electrocortical but not behavioral hyperarousal during the 3-day recording.

To determine if enhanced TDW results from more frequent wake-to-TDW transitions, or prolonged TDW, we quantified number and duration of TDW episodes. TDW bouts lasted markedly longer in DA^*OxR2-KO*^ mice during baseline dark, SD and recovery (KO vs CT; baseline dark: 12.8 ± 0.7 s vs. 8.7 ± 0.9 s; SD: 18.6 ± 1.7 s vs. 9.6 ± 1 s, recovery dark: 12.4 ± 0.7 s vs. 9.1 ± 0.7 s; Fig. 3c, bottom). Hence, during SD, mean TDW bout duration more than doubled. Baseline TDW bout duration distribution analysis showed that short-to-medium bout categories (4-64 s) were much rarer, while very long TDW episodes (≥17 min) were enhanced, resulting in *DA*^*OxR2-KO*^ mice spending 9.81 ± 4.35% of total TDW in this bout category, while DA^*OxR2-CT*^ did not display any 17 min-long TDW episode (*P* = 0.014, Mann-Whitney U statistics, Fig. 3d). Total number of TDW episodes, however, did not differ (Fig. 3c, top). Therefore, increased time spent in TDW is due to increased stability of the TDW state.

Analysis of TDW spectra of DA^*OxR1-KO*^ mice revealed lower delta (1-2.75 Hz) and slow-theta (6.75-8 Hz), but higher 8.5–10.75 Hz activity. DA^*OxR1&2-KO*^ mice show a similar pattern, with a more pronounced decrease in theta (Fig. 3e). In contrast, DA^*OxR2-KO*^ mice showed increased theta power (7.75–10.25 Hz; Fig. 3e). We then calculated the theta peak frequency in TDW of KO and CTs, which did not differ (Table S3), indicating that alterations in TDW theta power is not caused by shifts in TDW frequency. Together, these results indicate that mice lacking *Hcrtr2* in DA cells exhibit constitutive cortical activation, and spend more time in a brain state electrocortically akin to the one observed during exploratory behavior, even in the absence of external stimuli and accompanying locomotory changes. Combined dopaminergic HCRTR1&2 loss led to opposite changes, with higher infra-theta but lower theta, suggesting a state of hypoarousal, and divergent impacts of the two HCRTRs on dopaminergic circuits of arousal.

### Loss of *Hcrtr2* in dopaminergic neurons consolidates REMS

**Fig. 4:**
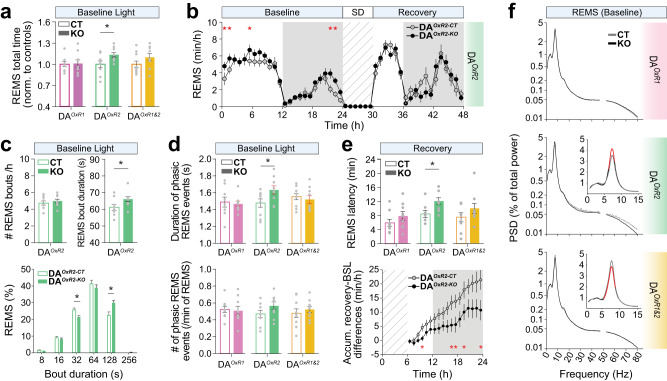
Dopaminergic *Hcrtr2*-ablated mice exhibit a prolonged and theta-enriched REMS. **a** Total time spent in REMS during baseline light period for DA^*OxR1-KO*^, DA^*OxR2-KO*^, and DA^*OxR1&2-KO*^ mice, normalized to their respective controls. DA^*OxR2-KO*^ mice spent more time in REMS in baseline light period than controls (*P* = 0.0341; independent *t*-test). **b** Time-course of REMS amount in DA^*OxR2-KO*^ and DA^*OxR2-CT*^ mice (two-way ANOVA; genotype effect F(1,23) = 14.208, *P* < 0.001; Bonferroni post-hoc test, *P < 0.05). **c** Number and duration of REMS bouts, and bout-duration distribution, during baseline light in DA^*OxR2-KO*^ and DA^*OxR2-CT*^ mice. *Top*, While number of REMS episodes did not differ between groups, mean duration of REMS bouts was increased in DA^*OxR2-KO*^ mice in baseline light (*P* = 0.0455, independent *t*-test). *Bottom*, Bars show the relative contribution of bout categories to total REMS time (%). DA^*OxR2-KO*^ mice spent a smaller fraction of REMS in bouts lasting 32–64 s, while they spent a larger fraction in bouts lasting 128-252 s, compared to controls (two-way ANOVA; genotypeXduration interaction F(8,153) = 5.165, *P* < 0.001; Tukey post-hoc test, *P < 0.05). Only the lower bin limit of bout durations is indicated for 8–12, 16–28, 32–60, 64–124, 128–252, >256 s long bouts. **d** Mean duration of phasic REMS events is longer in DA^*OxR2-KO*^ mice compared to controls (*P* = 0.0423, independent *t*-test). Number of phasic REMS events however is unchanged in all DA^*KO*^ models. **e** During recovery following SD, DA^*OxR2-KO*^ mice show increased REMS latency (*P* = 0.0167, independent *t*-test, Top), and a slower and less extensive REMS rebound (*Bottom*). REMS latency is unchanged in DA^*OxR1-KO*^ and DA^*OxR1&2-KO*^ mice. Time-course of REMS recovery after SD, calculated as accumulated excess time spent in REMS compared to baseline, shows that DA^*OxR2-KO*^ mice regain only ~40% as much REMS time compared to controls by end of the recovery dark phase (*P* = 0.007, independent *t*-test). **f** Baseline spectral profiles of REMS in DA^*OxR1-KO*^, DA^*OxR2-KO*^, and DA^*OxR1&2-KO*^ mice and respective controls. Power density values are expressed as % of total EEG power density. Red lines indicate significant differences. DA^*OxR2-KO*^ mice display enhanced REMS theta power (6.0–8.25 Hz) compared to controls (two-way ANOVA; genotypeXfrequency interaction F(266,4272) = 1.600, *P* < 0.001; Tukey post-hoc test, *P* < 0.05). In contrast DA^*OxR1&2-KO*^ mice show reduced theta power (5.5-8.75 Hz) compared to controls (two-way ANOVA; genotypeXfrequency interaction F(291,4964) = 1.251, *P* = 0.003; Tukey post-hoc test, *P* < 0.05). Bar graphs depict mean ± SEM. *n* = 9 mice/group, except n = 10 for DA^*OxR1&2-CT*^.

We next investigated the effects of HCRT → DA disruption on REMS architecture and oscillations. Total time spent in REMS was significantly prolonged in DA^*OxR2-KO*^ compared to controls in baseline light period (DA^*OxR2-KO*^: 65.7 ± 1.9 min *vs* DA^*OxR2-CT*^: 57.9 ± 2.8 min), but not in DA^*OxR1-KO*^ and DA^*OxR1&2-KO*^ mice (Fig. 4a, b). To determine whether enhanced REMS results from more frequent NREMS-to-REMS transitions, or from enhanced REMS stability, we quantified REMS episode number and duration. DA^*OxR2-KO*^ mice exhibited prolonged REMS bouts (DA^*OxR2-KO*^: 66.2 ± 1.5 s *vs* DA^*OxR2-CT*^: 61.2 ± 1.7 s), without changes in bout number (Fig. 4c, top). Analysis of bout duration distribution showed that, relative to controls, DA^*OxR2-KO*^ mice spent less of REMS in 32-64 s-long bouts, but more in 2–4 min-long bouts (Fig. 4c, bottom). Hence, loss of HCRT modulation of the DA system via HCRTR2 leads to both TDW and REMS state consolidation.

HCRT neurons are reported to discharge during phasic REMS [4], which represents intermittent rises in both amplitude and frequency of theta oscillations during REMS [49]. We therefore analyzed phasic REMS in our 6 genotype groups, and found that mean duration of these events is longer in DA^*OxR2-KO*^ relative to controls (DA^*OxR2-KO*^*:* 1.634 ± 0.05 s vs DA^*OxR2-CT*^: 1.478 ± 0.05 s; Fig. 4d), while it is unchanged in DA^*OxR1-KO*^ and DA^*OxR1&2-KO*^ mice.

A surprising finding was that DA^*OxR2-KO*^ mice were severely impaired in recovering REMS following SD. The homeostatic rebound in REMS was delayed and occurred at a slower rate (Fig. 4e, bottom). During the 18 h following SD, control mice engaged in a total of 23.8 ± 4 min of excess REMS compared to baseline, whereas DA^*OxR2-KO*^ mice’ REMS rebound only summed up to 9.0 ± 2.6 min. Therefore, although dopaminergic *Hcrtr2* inactivation prolongs REMS in baseline, REMS is diminished after a homeostatic challenge. NREMS rebound also tended to be reduced in DA^*OxR2-KO*^ mice during the post-SD dark phase (Fig. S5a, left**)**. These findings suggest involvement of dopaminergic HCRTR2 signaling in sleep homeostasis.

REMS spectral quality was next examined. We found opposing effects of single *Hcrtr2 vs* double receptor inactivation on theta power, which was significantly increased in DA^*OxR2-KO*^ mice across 6.0–8.25 Hz, but decreased in DA^*OxR1&2-KO*^ mice across 5.5–8.75 Hz (Fig. 4f). These changes strikingly parallel the ones observed during wakefulness and TDW, indicating that HCRT → DA modulation of theta power operates across states, in wakefulness, TDW and REMS.

### Enhanced theta-gamma coupling

**Fig. 5:**
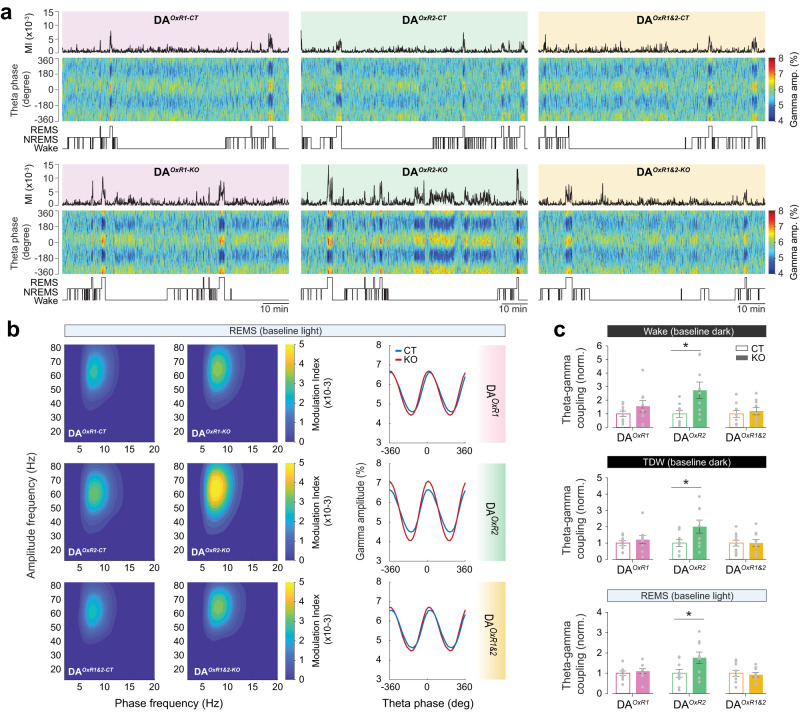
Dopaminergic *Hcrtr2*-ablated mice exhibit enhanced theta-gamma phase-amplitude coupling during both wakefulness and REMS. **a** Representative dynamics of theta-gamma coupling across vigilance states in DA^*OxR1-KO*^, DA^*OxR2-KO*^, and DA^*OxR1&2-KO*^ mice and respective controls during a 90-min interval in dark phase. Traces on top show the modulation index (MI) between theta (7–11 Hz) and fast-gamma (52–80 Hz) oscillations, calculated using a 4-s moving window. Heatmaps color-code the distribution of gamma amplitudes across the theta phase, i.e. depict phase-amplitude histograms of 4-s windows. Hypnogram is depicted below. **b** Heatmaps show the comodulogram analysis of phase-amplitude coupling for representative mice of each group during REMS (12-h baseline light). DA^*OxR1-KO*^ and DA^*OxR1&2-KO*^ mice show similar levels of theta-gamma coupling compared to their controls, while DA^*OxR2-KO*^ mice display enhanced coupling. Right panels show the phase-amplitude histograms of representative mice. **c** Pair-wise statistical comparisons between theta-gamma coupling values of KO and CT mice in wakefulness and TDW of baseline dark phase, and REMS of baseline light phase. Theta-gamma coupling significantly increased in DA^*OxR2-KO*^ mice compared to controls during all three states (DA^*OxR2-KO*^ vs DA^*OxR2-CT*^: wake: *P* = 0.0172; TDW: *P* = 0.0397; REMS: *P* = 0.0412; independent *t*-test). Bar graphs depict mean ± SEM. *n* = 9 mice/group, except *n* = 10 for DA^*OxR1&2-CT*^.

Theta and gamma oscillations are instrumental in cognition, and the strength of coupling between the theta phase and gamma amplitudes correlates with learning and task performance in rodents and humans [50, 51]. To investigate the role of dopaminergic HCRT signaling in modulating theta-gamma networks, we calculated phase-amplitude cross-frequency coupling between these two oscillations using the modulation index (Fig. 5, see Methods). We found that DA^*OxR2-KO*^ mice express higher theta-gamma coupling during waking and TDW in baseline dark phase (DA^*OxR2-KO*^ vs DA^*OxR2-CT*^; *P* = 0.0464; Fig. 5c), while theta-gamma coupling of DA^*OxR1-KO*^ and DA^*OxR1&2-KO*^ mice did not differ from controls. As theta and gamma frequencies also dominate the REMS EEG [52], we next measured theta-gamma coupling during REMS and found higher coupling index of the REMS theta rhythm of DA^*OxR2-KO*^ mice with gamma oscillations in baseline light phase (DA^*OxR2-KO*^ vs DA^*OxR2-CT*^; *P* = 0.0351; Fig. 5c), while *Hcrtr1* or *Hcrtr1&2* dopaminergic inactivation did not significantly affect REMS theta-gamma coupling.

### DA^*OxR2-KO*^ mice learn faster but exhibit maladaptive patterns of reward-seeking behavior

**Fig. 6:**
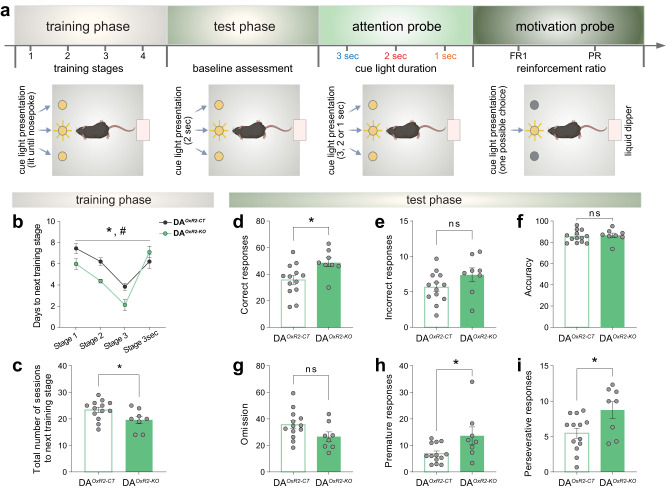
Dopaminergic *Hcrtr2*-ablated mice learn faster but show compulsive and impulsive-like behaviors. **a** Behavioral procedure by which mice perform a three-choice serial reaction time task (3-CSRTT), divided in training and test phases, and then undergo an attention and a motivation probe. The latter two probes are described in Fig. 7. **b** Training phase time-course. DA^*OxR2-KO*^ needed fewer days of training to reach the next stage of task acquisition compared to DA^*OxR2-CT*^ mice (two-way ANOVA, training effect: F(2.349, 44.63) = 27.15, *P* < 0.001, *genotype effect: F(1, 19) = 6.618, *P* = 0.0186, # interaction F(3, 57) = 3.708, *P* = 0.0166, with Sidak post-hoc test, *P* < 0.05). **c** Bar graphs depict the total number of sessions required to reach the next training stage. DA^*OxR2-KO*^ needed fewer sessions to reach the next stage of task acquisition compared to DA^*OxR2-CT*^ mice (*P* = 0.0404, independent *t*-test). During the test phase, DA^*OxR2-KO*^ mice displayed a higher number of correct responses (*P* = 0.0150, **d**), but no differences in incorrect responses (*P* = 0.1188, **e**), nor in response accuracy (*P* = 0.6737, **f**), or omissions (*P* = 0.0767, **g**) relative to DA^*OxR2-CT*^ mice. DA^*OxR2-KO*^ mice exhibited a higher number of premature responses, an index of impulsivity (*P* = 0.0286, **h**), and a higher number of perseverative responses, an index of compulsivity (*P* = 0.0203, **i**) compared to DA^*OxR2-CT*^ mice. Response accuracy was calculated as “correct responses/(correct+incorrect responses)x100”. Bar graphs depict mean ± SEM. *n* = 8 DA^*OxR2-KO*^, n = 13 DA^*OxR2-CT*^.

As DA^*OxR2-KO*^ mice present neurophysiological markers known to correlate with cognitive performance, e.g. increased alert wakefulness and higher theta-gamma coupling, we next compared executive control and reward sensitivity of DA^*OxR2-KO*^ and DA^*OxR2-CT*^ mice using an operant conditioning paradigm. Mice were trained in a 3-choice serial reaction time task (3-CSRTT) in which each mouse progresses through stages of increasing difficulty according to individual performance and proceeds to the next training stage when certain criteria are met (Fig. 6a and Methods). During the training phase, DA^*OxR2-KO*^ mice exhibited faster task acquisition, requiring fewer days (Fig. 6b), and fewer sessions (Fig. 6c), to reach the next training stage. When performance of all mice became stable on the same training contingencies, their performance during the last three sessions on the same stage was averaged (test phase). DA^*OxR2-KO*^ mice performed more correct responses compared to DA^*OxR2-CT*^ mice (Fig. 6d), with no difference in incorrect responses (Fig. 6e), nor in response accuracy (Fig. 6f), or number of omissions (Fig. 6g). However, DA^*OxR2-KO*^ mice showed more premature responses, i.e. responses preceding cue light illumination, suggesting an impulsive-like behavior (Fig. 6h), and more perseverative responses, i.e. repeated head entries in absence of reward intake, suggesting a compulsive-like trait (Fig. 6i). Collectively, these data show that DA^*OxR2-KO*^ mutants manifest improved performance in acquisition and maintenance of instrumental learning in a visual discrimination task, but they also concomitantly exhibit signs of impaired inhibitory control.

### DA^*OxR2-KO*^ mice show higher choice accuracy under increasingly demanding task contingencies

**Fig. 7:**
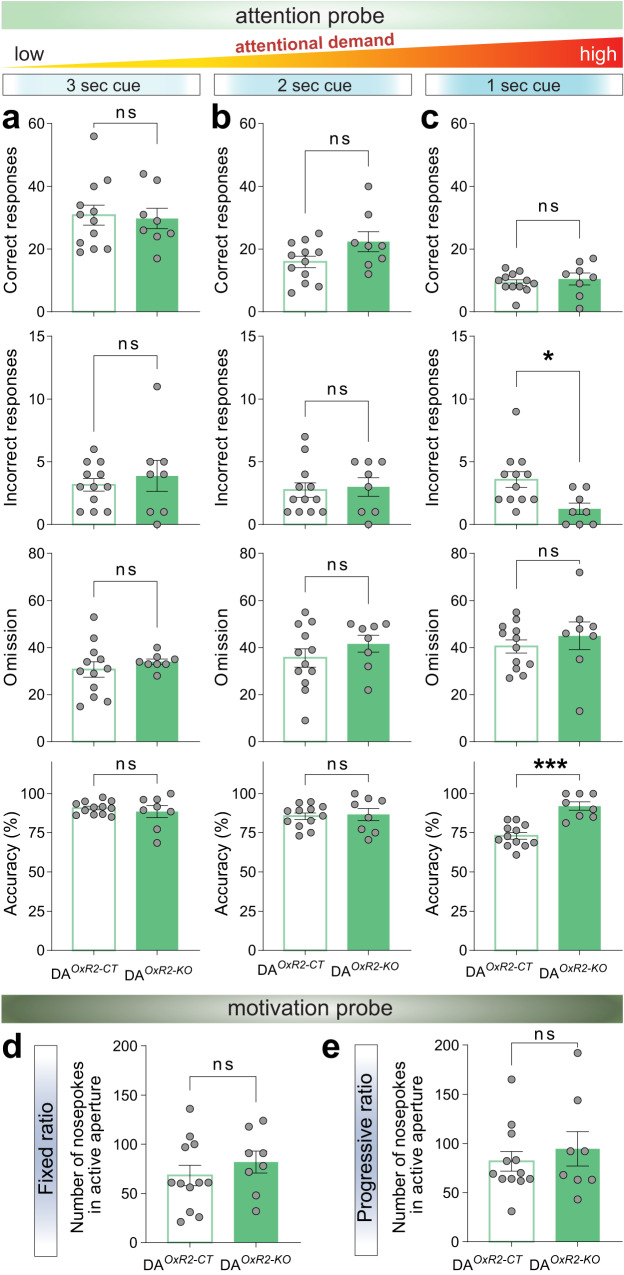
Dopaminergic *Hcrtr2*-ablated mice display higher choice accuracy under increasingly demanding task contingencies, but no alterations in motivational drive. Shown are results of the attention and motivation probes (see timeline in Fig. 6). In the attention probe, stimulus duration (i.e., cue light in apertures) varied from 3 to 1 s, thus exposing mice to contingencies of increasing attentional demand. **a** When the stimulus duration was 3 s, no differences between DA^*OxR2-KO*^ and DA^*OxR2-CT*^ mice were observed in correct and incorrect responses, omissions, and response accuracy (correct: *P* = 0.8213, incorrect: *P* = 0.5496, omission: *P* = 0.4297, accuracy: *P* = 0.5141, independent *t*-test). **b** Similarly, no differences were observed when the stimulus duration was 2 s (correct: *P* = 0.0739, incorrect: *P* = 0.7905, omission: *P* = 0.2969, accuracy: *P* = 0.8025, independent *t*-test). **c** When the stimulus duration was reduced to 1 s, no differences were observed between genotypes regarding correct responses and omissions (correct: *P* = 0.5486, omission: *P* = 0.4516, independent *t*-test). However, DA^*OxR2-KO*^ mice displayed fewer incorrect responses, and a higher response accuracy (incorrect: *P* = 0.0133, accuracy: *P* < 0.001, independent *t*-test). In the motivation probe, a single aperture was illuminated and only this choice was rewarded. Mice were challenged on a fixed ratio on one day, and on a progressive ratio on the next day. DA^*OxR2-KO*^ and DA^*OxR2-CT*^ mice did not show significant differences in number of nosepokes in the active aperture under a fixed ratio (*P* = 0.3926, independent *t*-test, **d**), nor the next day under a progressive ratio (*P* = 0.5083, independent *t*-test, **e**). Bar graphs depict mean ± SEM. *n* = 8 DA^*OxR2-KO*^, n = 12 DA^*OxR2-CT*^.

We next examined whether faster task acquisition during the training phase and enhanced correct responses during the test phase in DA^*OxR2-KO*^ mice, could be denoting increased motivational drive rather than improved attentional skills. We performed two additional experiments that separately address aspects of attentional performance and motivation, in a cognitive effort, and a physical effort-demanding task, respectively (Fig. 6a). To assess attentional performance, we progressively decreased the duration of the light cue indicating the correct nosepoking response, assessing the mice with the cue lasting 3, 2 and 1 s, i.e. under increasing attentional demands. No differences were observed between genotypes when the cue light was 3 or 2 s in correct and incorrect responses, omissions, or accuracy (Fig. 7a, b). However, under the most difficult condition with the cue lasting 1 s, DA^*OxR2-KO*^ mice showed higher performance reflected by fewer incorrect responses and concomitant increased accuracy (Fig. 7c), with no differences in number of correct responses, or omissions. These data suggest that DA^*OxR2-KO*^ mice have an enhanced capability to sustain attention, and perform with higher accuracy when attentional demands are increased, and align with the hypervigilance reported above.

To further disentangle increased cognitive performance from reward-based invigoration [53] or motivational drive, we next subjected mice to a fixed and progressive schedule of reinforcement to evaluate their willingness to work for the reward. To minimize the contribution of attentional load, now only one hole was illuminated. There was no difference between DA^*OxR2-KO*^ and DA^*OxR2-CT*^ mice in number of nosepoking responses, neither under a fixed (Fig. 7d), nor progressive ratio (Fig. 7e), suggesting no alterations in motivation in an effortful task. Overall, these results demonstrate improved performance of DA^*OxR2-KO*^ mice when attentional demands are high, namely during initial task acquisition, and when task contingencies are more demanding, but they also show maladaptive patterns of reward-seeking behaviors when assessed under well-trained conditions.

## Discussion

Our data establish a genetically-defined link between monosynaptic HCRT → DA connectivity and theta oscillatory power in wakefulness and REMS, and furthermore identify a link between HCRT → DA neurotransmission and executive functioning. We disconnected the dopaminergic system from HCRT input by selective *Hcrtr1* or *Hcrtr2* deletion in DA neurons. Although DA^VTA^ cells are the best characterized dopaminergic HCRT target, other potential HCRT target DA groups in hypothalamus or dorsal raphe would also be disconnected from HCRT input. Loss of *Hcrtr2* caused dramatic increases in waking theta activity and time spent in alert wakefulness, albeit uncoupled from locomotion, although EEG theta is commonly associated with locomotion [42]. Thus DA^*OxR2-KO*^ mice show electrocortical hyperarousal, but not evident behavioral hyperarousal as evaluated by locomotor activity in the homecage environment. Locomotor responses in novel contexts remain however to be evaluated. The increased rates of premature and repetitive (perseverative) responding in the 3-CSRTT test chamber suggest that behavioral hyperactivity is present.

REMS was also affected, with stronger theta activity, longer bouts, and total duration. Furthermore, fast-gamma amplitudes showed higher coupling to the theta phase during wakefulness and REMS. Stronger theta-gamma coupling was associated with cognitive advantages, namely superior learning speed and attentional performance, however compromised by maladaptive patterns of reward-seeking, impulsivity and compulsivity-like behaviors. DA^*OxR2-KO*^ mice therefore earned more rewards, but presumably at a higher energetical cost. Disconnection between learning, attentional, and executive skills is intriguing and requires investigation in additional contexts. None of the EEG changes observed in DA^*OxR2-KO*^ mice were seen in mice with chronic disruption of *Hcrtr1*, or both receptors, in DA cells. Strikingly, DA^*OxR1&2-KO*^ mice exhibited opposite changes, with decreased theta activity in wakefulness, TDW and REMS. Our results thus demonstrate critically distinct neuromodulation of DA pathways by *Hcrtr1* and *Hcrtr2*, with a crucial role of *Hcrtr2* in theta/gamma oscillations and associated cognition.

Although implication of the HCRT → DA circuit in theta is novel, ample evidence exists for modulation of theta by HCRT and DA individually. We previously reported profound disruption of theta in *Hcrt*-KO mice, which show a severely theta-depleted baseline wakefulness, through inability to maintain TDW for extended periods [45, 54]. TDW instability of *Hcrt*-KO mice phenocopies excessive daytime sleepiness of narcolepsy patients, who typically fail to maintain arousal when under-stimulated. Similar to patients, when subjected to external stimulation, *Hcrt*-KO mice showed normalized TDW maintenance [45]. This is in strike contrast to DA^*OxR2-KO*^ mice, which display increased TDW across spontaneous or enforced wakefulness, a priori challenging our understanding of HCRT signaling.

HCRT neurons are well positioned to influence hippocampal theta as they profusely innervate the medial septum (MS), a structure which expresses *Hcrtr2* RNA [55], and projects to the hippocampus and paces hippocampal theta bursting activity. Destruction of HCRT-2-binding neurons in MS of rats dramatically decreased theta during wakefulness and REMS [56]. DA^VTA^ axons also project to the septum and contribute to regulate theta oscillations [57]. Hence both HCRT and DA^VTA^ project to the septum and regulate hippocampal theta during wakefulness and REMS.

A large body of data points to DA as facilitator of theta activity [58, 59]. DA agonists and NMDA-glutamatergic DA^VTA^ activation induce theta, while VTA silencing or lesioning disrupts theta [60–62]. Prominent hippocampal theta appears in two states, active wakefulness (AW) and REMS, both featuring powerful DA^VTA^ bursting activity [27]. The theta peak frequencies in VTA and hippocampal local field potentials are highly inter-correlated, during AW and REMS [27, 57]. Since AW and REMS are both high-cholinergic states sustained by pedunculopontine/laterodorsal tegmental nuclei (LDT/PPT) activity, and LDT/PPT send excitatory input to DA^VTA^ cells [63], the DA^VTA^ system may belong to a theta-generating AW and REMS-active LDT/PPT → DA^VTA^ → MS→hippocampal circuit [57].

Although generally associated, DA activity and theta power in some contexts are inversely related, e.g. permanent hyperdopaminergia in *Dat*-KO mice is associated with decreased hippocampal theta [64], and DA-depleted rats show theta amplification during cognitive tasks [65]. Thus manipulations of the DA system may bidirectionally modulate downstream effectors, due to heterogeneities in DA subpopulations’ electrophysiological properties, receptive fields, projections, or in the subclass/distribution of receptors. For instance, DA agents can show inverted-U-shaped dose responses, e.g. too little or too much DA impairs working memory. D2/3-agonists enhance sleep at low dosage, but wakefulness at higher doses [66], depending on whether presynaptic or postsynaptic effects dominate.

Interestingly, continued DA^VTA^ optogenetic stimulation induces a state of theta-enriched wakefulness [28] closely resembling baseline wakefulness of DA^*OxR2-KO*^ mice. Although optogenetic activation and genomic manipulation are not directly comparable, dopaminergic *Hcrtr2* loss may likewise cause sustained theta activity and dopaminergic activation. Genetic effects at two loci can be non-additive, and the opposite effects of dual *Hcrtr1&2* vs single *Hcrtr2* dopaminergic disruption, with *Hcrtr1&2* most resembling *Hcrtr1*, suggests that *Hcrtr1* is epistatic to *Hcrtr2* in DA neurons. Nevertheless, our findings’ simplest interpretation remains that DA^*OxR2-KO*^ mice increased theta reflects enhanced DA activity during wakefulness and REMS, while HCRTR2 signaling normally dampens theta networks via dopaminergic inhibition. HCRT is usually excitatory, however inhibitory HCRT-2 activity via HCRTR2 is reported [67–69]. Conversely, since DA^*OxR1&2*^-doubly-deficient mice experience reduced theta in waking and REMS, paired dopaminergic HCRTR1&2 activity may normally stimulate DA cell activity and theta networks. Consistent with this hypothesis, DA^*OxR1&2-KO*^ mice display beta enhancement, which is a marker of DA deficiency. Beta activity is normally repressed during movement, and its pathological increase is characteristic of PD and DA-depleted rats [47, 70]. Interestingly, HCRT-1 and HCRT-2 are neuroprotective in PD animal models [71], underscoring the potential importance of HCRT → DA neurotransmission in PD, and relevance of DA^*OxR1&2-KO*^ mice as model of DA-deficiency relevant to PD.

Disrupting HCRT → DA circuits impacted REMS and wakefulness almost identically. As mentioned, HCRT neurons show bursting activity during phasic REMS [4, 72], and a sublaterodorsal tegmental nucleus-projecting REMS-active HCRT population was shown to sustain theta and prolong REMS [7]. Consistent with active HCRT → DA signaling in phasic REMS, DA^*OxR2-KO*^ mice exhibit prolonged phasic REMS events. An increasing number of studies also evidence DA activity during REMS. DA^VTA^ neurons show increased *c-Fos* expression during REMS rebound [73], and NAc and medial prefrontal cortex (mPFC) DA release increases in REMS [74]. DA^VTA^ single-unit recording in rats revealed prominent burst firing during REMS, as during palatable food consumption [27], and DA^VTA^ activity increases at NREMS-to-REMS transitions [28]. Conversely, DA-depleted rats do not enter REMS, which is restored by D2-agonists [75]. A recent study positioned DA^VTA^ cells as prime inducers of REMS, demonstrating DA transients appearing within the BLA ~20 s before REMS-onset, while optogenetic activation of BLA DA^VTA^ terminals induced REMS [76]. In agreement, DA^VTA^ bursting activity observed by unit-recordings appears ~10-20 s before REMS-onset [27].

An unexplained DA^*OxR2-KO*^ phenotype is a strongly diminished REMS rebound following SD. Could the shorter REMS rebound of DA^*OxR2-KO*^ mice stem from REMS higher theta power, whereby enhanced theta synchrony would facilitate the efficiency of REMS-dependent recovery processes? REMS homeostasis is poorly understood. The physiological and cognitive processes of REMS may rely on the combined action of prominent cholinergic and dopaminergic neuromodulation [27], and our study further suggests involvement of HCRT → DA transmission in sleep recovery.

A 3rd theta-dominated state is cataplexy, narcolepsy pathognomonic symptom and HCRT deficiency signature. D2/D3-receptors modulate cataplexy in narcoleptic dogs and mice, with D2/D3-agonists aggravating, and blockers improving cataplexy [77, 78]. Because D2/D3 are DA neuron inhibitory auto-receptors, this suggests that DA insufficiency precipitates cataplexy. Optogenetic excitation of DA^VTA^ terminals within BLA of narcoleptic mice exposed to chocolate induced DA transients and cataplexy, while WT mice experienced a much smaller DA release [76], suggesting HCRT may inhibit DA release. Accordingly, our HCRT → DA-deficient mice may display cataplexy upon chocolate consumption. We did not evidence cataplexy in our mice, but they were not exposed to chocolate or other potent triggers. The role of HCRT → DA circuits in cataplexy warrants further investigation.

Theta-gamma coupling facilitates, or reflects, short-term memory processes. Rats learning day-by-day to associate contexts with food location, demonstrated increasing theta-gamma coupling as learning progressed, and theta-gamma coupling could predict the probability of correct choice on a given day [50]. Accordingly, DA^*OxR2-KO*^ mice’ increased coupling is expected to enhance learning speed and response accuracy, two expectations that we confirmed in the 3-CSRTT. DA^*OxR2-KO*^ mice not only learnt the task faster, but they reached higher accuracy at baseline, and when attentional demand increased, performed better. The mPFC is a major DA^VTA^ target, as well as a direct HCRT target, and a hub for executive functions. DA^*OxR2-KO*^ mice may experience malfunction of a HCRT → DA^VTA^ → mPFC circuit [79] resulting from deficient HCRTR2 signaling in DA cell bodies or terminals. Lambe et al. demonstrated that HCRTR2 signaling at PFC thalamocortical terminals plays a role in executive functions, whereby intra-PFC HCRT-2 infusion improved high-attention-demanding task accuracy, by exciting thalamocortical terminals onto layer V pyramidal cells [80]. Importantly, HCRT can act either by postsynaptic or presynaptic action, i.e. acting on cell somata, or axon terminals. In terminals, HCRT can modulate release of neurotransmitters, e.g. glutamate, GABA, and potentially DA [36, 37, 81, 82]. Deficient HCRTR2 signaling in DA^VTA^ terminals, that densely innervate the PFC and co-release DA and glutamate [83], may thus contribute to DA^*OxR2-KO*^ mice’ phenotype.

DA^*OxR2-KO*^ mice enhanced performance was compromised by premature responding, aligning with studies implicating HCRT in impulsivity [84], and with observation that intra-VTA HCRTR1&2-dual-antagonist application reduces cocaine-evoked premature responding in rats [85]. DA^*OxR2-KO*^ mice exhibited moreover perseverative responding, with repeated head entries after reward intake. Both premature and repetitive responding suggest behavioral hyperactivity. Altogether, DA^*OxR2-KO*^ mice phenotype depicted several endophenotypes reminiscent of the neurodevelopmental disorder attention deficit/hyperactivity disorder (ADHD), characterized by inattention, impulsivity, and hyperactivity symptoms, which can exist together or in isolation. Firstly, DA^*OxR2-KO*^ mice waking EEG revealed constitutive electrocortical hyperarousal, with increased theta/fast-gamma power. Enhanced waking theta is documented in children with ADHD and ADHD mouse models [86, 87]. Second, our mice display impaired inhibitory control, with marked impulsivity. In the visual task we used, impulsivity appeared coupled to enhanced attention. Three ADHD subtypes are distinguished, subtype-H (hyperactive), -I (inattentive)- and -C (combined). DA^*OxR2-KO*^ mice may feature an ADHD-H endophenotype, although other attentional modalities need appraisal in our mice. DA^*OxR2-KO*^ mice also show strong compulsivity, a trait over-represented in ADHD [88]. Genetic and pharmacological evidence strongly suggest involvement of DA pathways in ADHD in humans and mouse models. The ‘DA hypothesis of ADHD’ posits that DA hypofunction, or imbalance, underlies ADHD [89, 90]. Meanwhile, HCRT deficiency is also linked to ADHD. Narcolepsy patients show impaired executive function and impulsivity [91], and clinical overlap exists between narcolepsy and ADHD [92]. Suggesting shared causalities, narcolepsy is associated with increased ADHD incidence [93, 94]. Furthermore, both pathophysiologies respond favorably to DA drugs. Whereas methylphenidate and amphetamine cause hyperarousal in normal subjects, they normalize arousal in ADHD patients, and improve sleepiness in narcolepsy. Hence both HCRT and DA are established candidate ADHD substrates, and our study suggests that HCRT → DA connectivity is especially relevant.

Owing to their vital role in neuroplasticity, learning and memory, theta and gamma oscillations and their phase coupling are cognitive biomarkers and therapeutic targets. Oscillatory alterations are observed in neuropsychological disorders, including arousal disorders, anxiety, and depression. Stimulants and other therapeutic interventions induce EEG changes that correlate with clinical benefits. Oscillations therefore can act as non-invasive biomarkers of recovery. Understanding circuits governing theta/gamma neuromodulation may moreover lead to disease-causing mechanisms. Post-mortem quantitative studies evidenced five-fold heavier HCRT input to TH-immunoreactive neurons in human VTA as compared with rat [95]. Hence, HCRT signaling in VTA may play as critical a role in reward processing and cognition in humans as it does in rodents, and further understanding of HCRT → DA connectivity may guide novel therapies.

## Materials and Methods

### Creation of *Hcrtr2* conditional knockout mice

*Hcrtr1*^*flox*^ mice generation was previously described [38, 96]. *Hcrtr2*^*flox*^ allele creation is detailed in SI Appendix. The allele is designed so the *Hcrtr2* promoter drives *Gfp* instead of *Hcrtr2* following Cre/lox-recombination. Lox-lox recombination deletes DNA encoding HCRTR2 signal peptide, N-terminal domain, and almost entire transmembrane-domain1. To create a full-body *Hcrtr2*-null/GFP-reporter allele, *Hcrtr2*^flox^ mice were crossed to *Tg*(*EIIa*-*cre*)*C5379Lmgd* mice, which express Cre in the early embryo [97], producing *Hcrtr2*^*del*^ mice. To functionally validate *Hcrtr1* and *Hcrtr2* gene engineering, *Hcrtr2*^*del*^ were crossed with *Hcrtr1*^*del*^ mice [38, 98], and double-KO (*Hcrtr1*^*del*/del^,*Hcrtr2*^*del/del*^) were demonstrated to display narcolepsy with cataplexy by EEG/EMG-video analysis [13].

### Animals

Animal husbandry is detailed in SI Appendix. All animal procedures followed Swiss federal laws and were approved by the State of Vaud Veterinary Office. Care was taken at all times to optimize wellbeing and minimize discomfort and stress.

### Three-choice serial reaction time task (3-CSRTT)

The test is detailed in SI Appendix. Briefly, mice were trained to self-administer a 0.2% saccharine liquid reward. A nosepoke in the ‘active’ port activated delivery of 0.01 ml reward from a liquid dipper. The test comprised four increasing-difficult training stages and a test phase. To advance to the next stage, mice had to earn a fixed number of rewards during a 30-min session. Test phase performance was the average performance during the last 3 days’ sessions. Premature, correct, incorrect, omission, and perseverative responses were recorded. A premature response is nosepoking before cue light illumination. A perseverative response is a supernumerary head entry in liquid dispenser after reward consumption.

### Supplementary information

2023MP000713RR_SI

## Data Availability

The datasets acquired for this study are available from the corresponding author upon request.

## Code availability

Custom-written scripts for data analysis used in this study are available from the corresponding author upon request.
