# Supplementary material for: Inactivation of hypocretin receptor-2 signaling in dopaminergic neurons induces hyperarousal and enhanced cognition but impaired inhibitory control
Source: Mol Psychiatry. 2023 Dec 21;29(2):327–41. doi: 10.1038/s41380-023-02329-z (PMC11116111; doi:10.1038/s41380-023-02329-z)
Supplement: Supplementary file 1 — 2023MP000713RR_SI [file 41380_2023_2329_MOESM1_ESM.pdf]

Supplementary Information

Inactivation of hypocretin receptor-2 signaling in dopaminergic neurons induces hyperarousal and enhanced cognition but impaired inhibitory control

Mojtaba Bandarabadi[1†], Sha Li[1†], Lea Aeschlimann[2], Giulia Colombo[3], Stamatina Tzanoulinou[1], Mehdi Tafti[1], Andrea Becchetti[3], Benjamin Boutrel[2], and Anne Vassalli[1*]

[1]Department of Biomedical Sciences, University of Lausanne, Lausanne, Switzerland.
[2]Centre for Psychiatric Neuroscience, Department of Psychiatry, The Lausanne University Hospital, Lausanne, Switzerland.
[3]Department of Biotechnology and Biosciences, University of Milano-Bicocca, Milano, Italy.

[†]These authors contributed equally to this work.

**Corresponding author Email: Anne.Vassalli@unil.ch*

Supplementary Information includes:

Figures S1 to S8
Tables S1 to S3
SI Text (Materials and Methods)
SI References

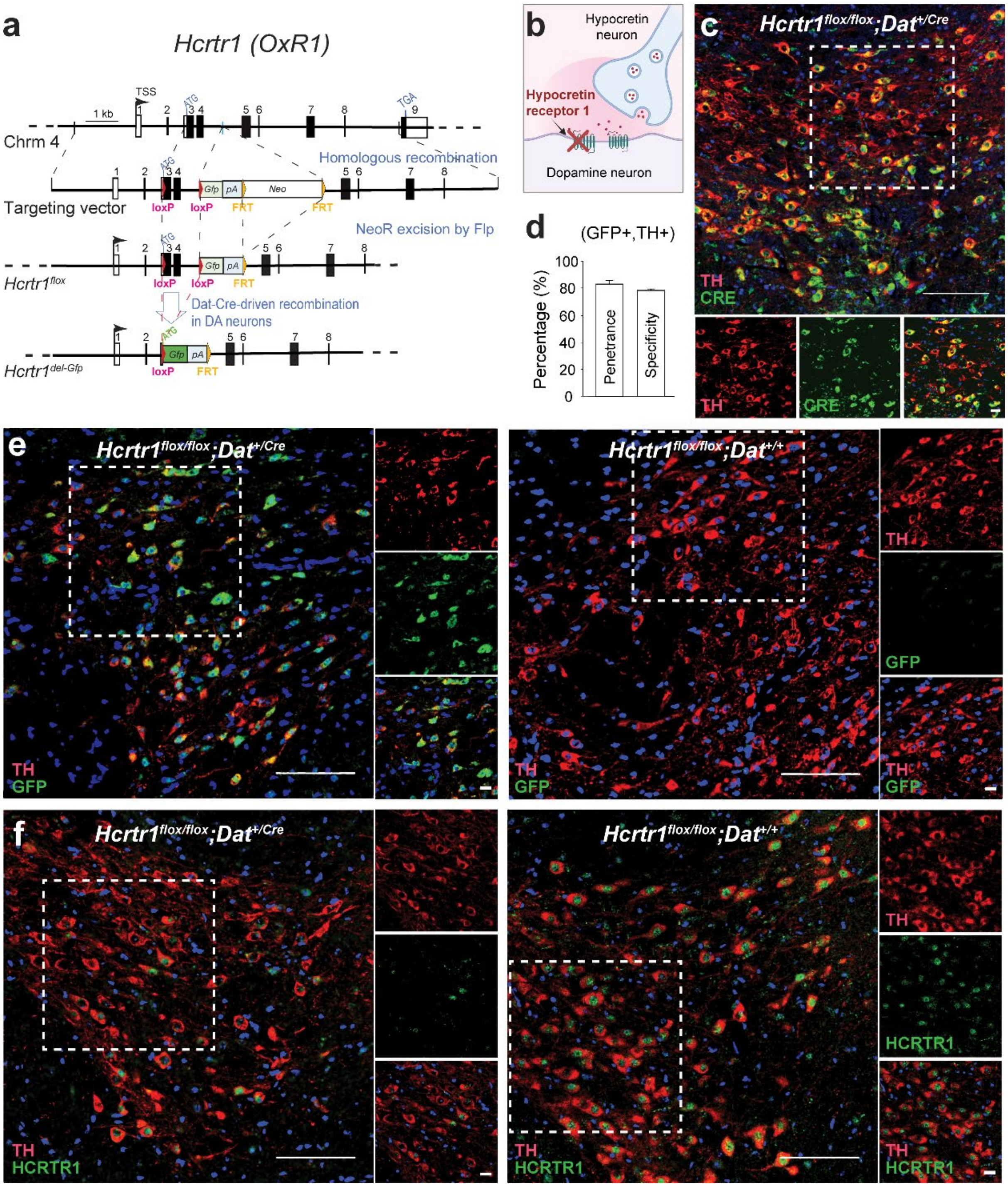

Figure S1: Generation of mice with dopamine neuron-specific *Hcrtr1* gene disruption.

(**a**) Schematic representation of homologous recombination between the *Hcrtr1* genomic locus and the targeting vector. Two loxP sites (red triangles) were inserted to flank the first coding exon (exon 3) and exon 4, which together encode the N-terminal first 126 aa of HCRTR1. The neomycin resistance gene used for selection in embryonic stem cells (neo; flanked by two FRT sites shown as orange triangles) was deleted using FLP recombinase, creating the *Hcrtr1^{flox}* allele. In *Dat-IRES-Cre*-expressing cells, CRE excises the inter-loxP segment (1.1 kb), creating the *Hcrtr1$^{del\text{-}Gfp}$* allele. The ATG translation start codon of *Hcrtr1* is replaced by the ATG of *Gfp*, and the *Hcrtr1* gene promoter now drives expression of *Gfp* (green rectangle), instead of *Hcrtr1*. *Gfp* reading frame is followed by a polyadenylation site (pA) to terminate transcription and prevent downstream expression. Boxes

depict exons (dark: protein-coding sequences, white: untranslated regions). TSS, transcription start site.

(**b**) Schematic representation of DA$^{OxR1\text{-}KO}$ mice.

(**c**) Representative confocal images of the the VTA of DA$^{OxR1\text{-}KO}$ mice subjected to fluorescence immunostaining using Tyrosine Hydroxylase (TH) and CRE antibodies evidence efficient expression of Dat-IRES-Cre by TH$^+$ neurons. (CRE$^+$, TH$^+$) cell quantification to evaluate penetrance and specificity of CRE expression in DA neurons is shown in Supplementary Figure 2.

(**d**) (GFP$^+$, TH$^+$) cell quantification demonstrates efficiency of *Hcrtr1* Exons 3-4 deletion in DA cells of the ventral midbrain (-2.92 to -3.88 mm from bregma) of DA$^{OxR1\text{-}KO}$ mice, with an overall penetrance (% of TH$^+$ neurons co-expressing GFP) of 83.0±2.8 % (n=12 sections, 2 mice), and a specificity (% of GFP cells co-expressing TH) of 74.4±0.5 (n=12 sections, 2 mice).

(**e,f**) Representative confocal images of the VTA of mice of the indicated genotypes that were subject to fluorescence immunostaining using the indicated antibodies. (e) *Dat*-driven Cre mediates DA-selective loxP site recombination and replacement of *Hcrtr1* coding sequences with *Gfp*, as shown by TH and GFP co-immunoreactivity in DA$^{OxR1\text{-}KO}$ (left), but not DA$^{OxR1\text{-}CT}$ (right) VTA. (f) Consistently, VTA TH$^+$ cells of DA$^{OxR1\text{-}CT}$ mice express HCRTR1 immunoreactivity (right), while VTA TH$^+$ cells of DA$^{OxR1\text{-}KO}$ littermates do not (left). Scale bar, 100um in large field images, and 20um in small field images. All images were taken at 40x magnification. *Hcrtr1*^{flox} is *Hcrtr1*$^{tm1.1Ava}$ (MGI:5637400), and *Hcrtr1*$^{KO\text{-}Gfp}$ is *Hcrtr1*$^{tm1.2Ava}$ (MGI: 5637401).

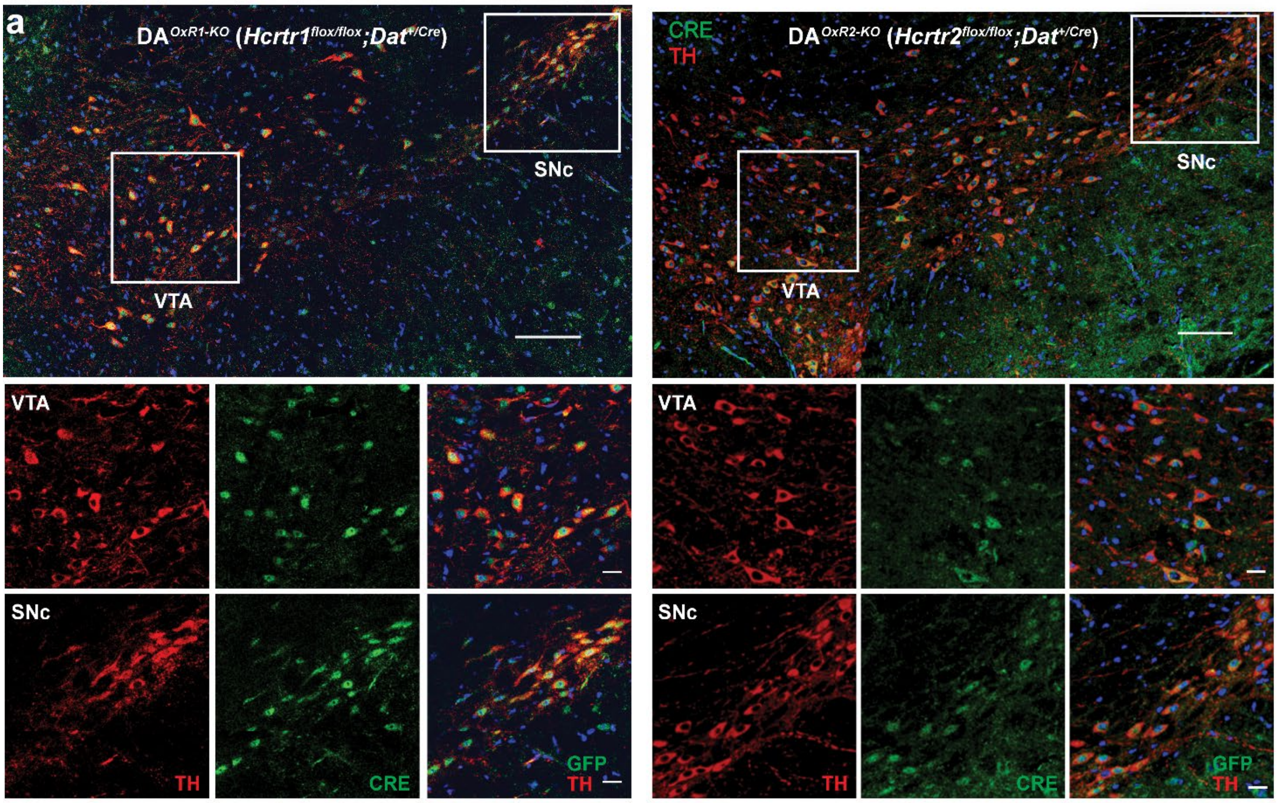

b DA$^{OxR1-KO}$, DA$^{OxR2-KO}$

Total TH+ cells (ave cell# per mouse, n=4)	Total CRE+ cells (ave cell# per mouse, n=4)	TH+, CRE+ cells (ave cell# per mouse, n=4	Penetrance % co-expression/TH cells	Specificity % co-expression/CRE cells
663	696	585	88.4 ± 1.1	84.4 ± 1.2

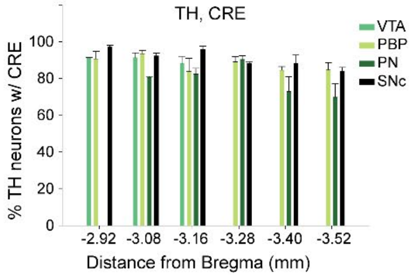

Figure S2: CRE immunostaining demonstrates the efficiency and specificity of the *Dat-IRES-Cre* allele to target dopamine neurons.

(**a**) Representative confocal images. Coronal sections of the ventral midbrain of DA$^{OxR1-KO}$ and DA$^{OxR2-KO}$ mice were subject to immunofluorescence staining using anti-Tyrosine Hydroxylase (TH, red) and anti-CRE (green) antibodies to verify signal co-localization.

(**b**) Neuronal counts throughout VTA and SNc showed that 88.4±1.1 % of TH-immunoreactive cells were CRE-positive (efficiency) and 84.4±1.2 % of CRE-immunoreactive cells were TH-positive (specificity) (n=4 mice). VTA, ventral tegmental area; SNc, Substantia nigra pars compacta.

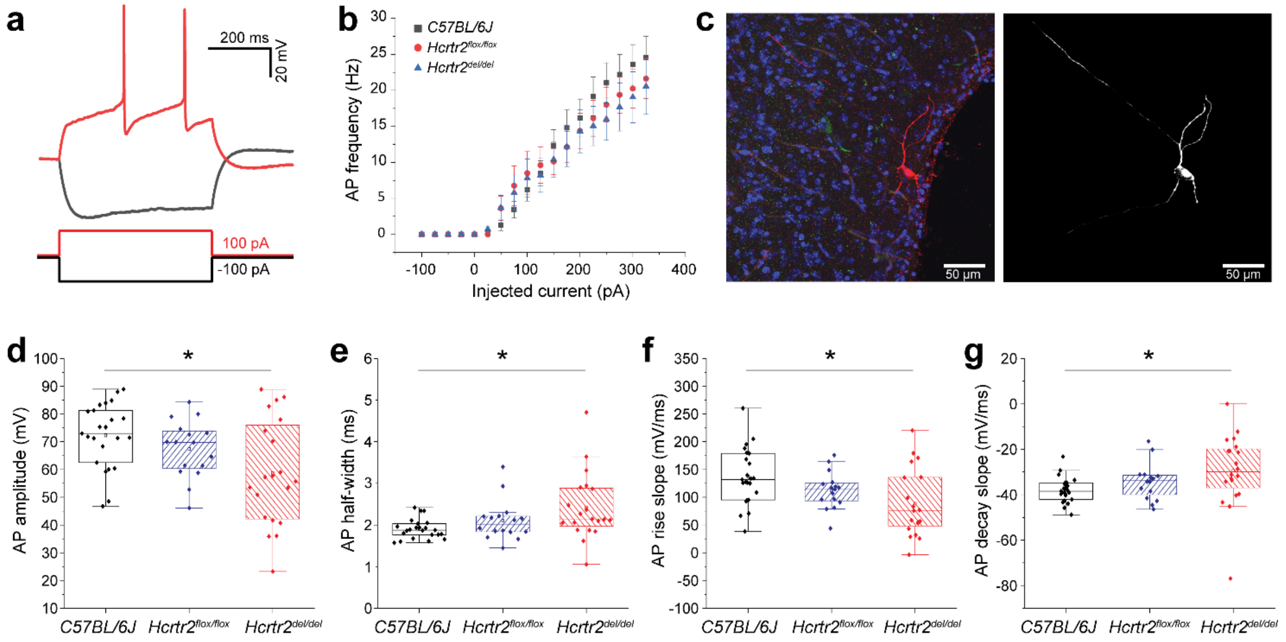

Figure S3: Identification and electrophysiological features of TMN histaminergic (HA) neurons.

(**a**) Putative HA neurons were characterized by testing the electrophysiological response to consecutive 25 pA current steps, lasting 500 ms, ranging from -100 to +325 pA. Typical responses to -100 pA and -100 pA stimulations show, respectively, the classical I_h-dependent sag on hyperpolarization, and the low-frequency late firing.

(**b**) Relation between injected current and firing frequency for putative histaminergic neurons from *C57BL/6J*, $Hcrtr2^{flox/flox}$ and $Hcrtr2^{del/del}$ mice, respectively). Data points are average firing frequencies calculated from a coherent ensemble of neurons and plotted as a function of injected current. Passive membrane properties are coherent with those reported by Haas and Reiner[1] (STable 1).

(**c**) Post-recording morphological reconstruction of a biocytin-loaded TMN neuron from a $Hcrtr2^{flox/flox}$ mouse, confirming the large diameter (20-30 µm) and multipolar shape of TMN HA neurons. Left: Maximal intensity projection of a 40x Z-stack of a 300 µm slice processed for IHF against biocytin and DAPI. Right: Projection of the 3D reconstruction. Scale bar 50 µm.

(**d-g**) AP features of *C57BL/6J* and $Hcrtr2^{flox/flox}$ neurons are also in line with prior reports, but $Hcrtr2^{del/del}$ cells displayed a reduced mean and more variable AP amplitude (d, P=0.011), an increased mean and more variable AP half-width (e, P=0.013), a reduced AP rise slope (f, P=0.010) and an increased AP decay slope (g, P=0.043). These slightly slower AP dynamics suggest that maturation of TMN HA cells in $Hcrtr2^{del/del}$ mice may be partly compromised, pointing to a potential role of HCRTR2 in the developmental specification of the HA neuronal phenotype. In fact, AP amplitude and duration are known to respectively increase and decrease during differentiation of cell types such as Cajal-Retzius cortical layer I neurons[2] and CA1 pyramidal neurons[3, 4]. This hypothesis warrants further investigation, to decipher the potential role of HCRTR2 in neuronal maturation.

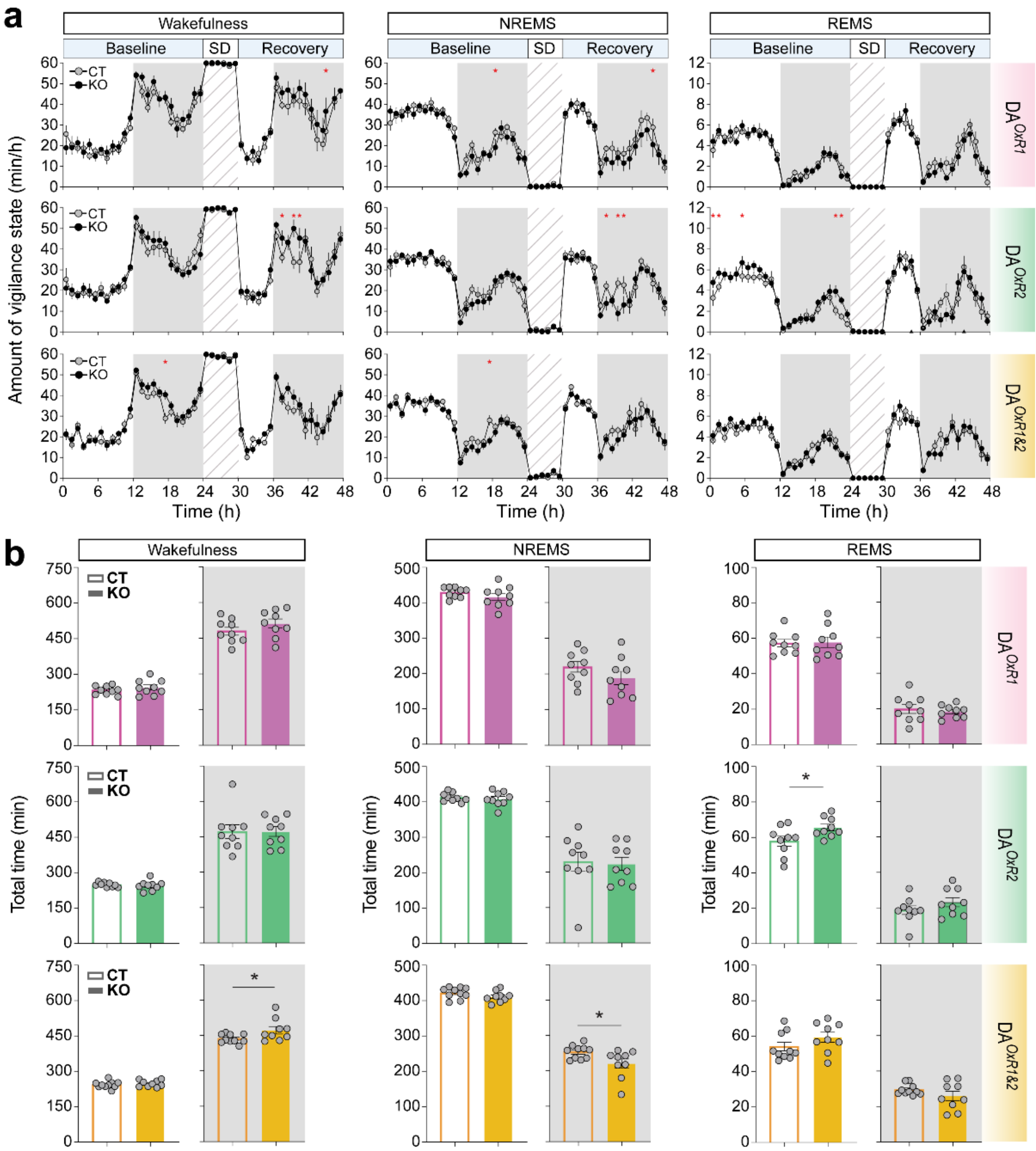

Figure S4: Vigilance states quantification in three HCRT-to-DA mutant mouse models.

(**a**) Hourly amounts of each state are given in minutes across the baseline, SD, and recovery recordings. Values are hourly means±SEM in min/h. Sleep/wake rhythmicity in baseline conditions was normal in all genotypes, albeit KO mice show increase in waking time in limited intervals of the active phase relative to controls (KO vs CT, two-way ANOVA, genotype effects; wake: $DA^{OxR1\text{-}KO}$: recovery: $F(1,23)=6.944$, $P=0.009$; $DA^{OxR2\text{-}KO}$: recovery: $F(1,23)=4.109$, $P=0.043$; $DA^{OxR1\&2\text{-}KO}$: baseline: $F(1,23)=8.482$, $P=0.004$; NREMS: $DA^{OxR1\text{-}KO}$: baseline: $F(1,23)=6.234$, $P=0.013$; recovery: $F(1,23)=6.924$, $P=0.009$; $DA^{OxR2\text{-}KO}$: recovery: $F(1,23)=5.109$, $P=0.024$; $DA^{OxR1\&2\text{-}KO}$: baseline: $F(1,23)=11.812$, $P<0.001$; REMS: $DA^{OxR2\text{-}KO}$: baseline: $F(1,23)=16.516$, $P<0.001$; Bonferroni post-hoc test, $^{*}P<0.05$).

(**b**) Histograms show total amount of wake, NREMS and REMS in baseline light and dark periods ($^{*}P<0.05$, independent *t*-test). n=9 mice per group, except n=10 for $DA^{OxR1\&2\text{-}CT}$.

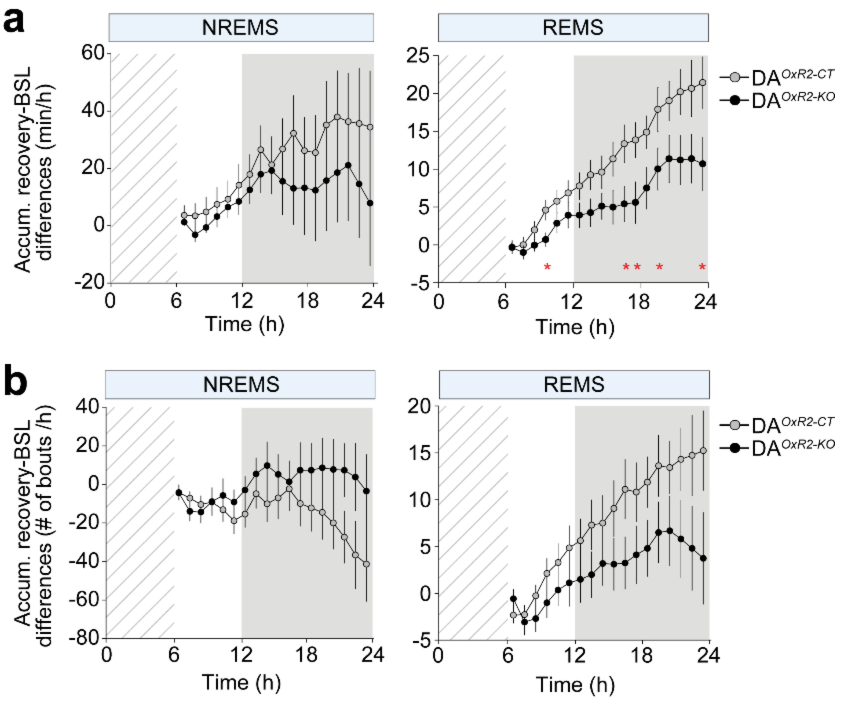

Figure S5: Quantification of NREM and REM sleep recovery following sleep deprivation in dopaminergic *Hcrtr2*-ablated mice.

(a) Time-course of NREMS (left), or REMS (right), recovery after SD, expressed as accumulated excess time spent in each state compared to baseline (min/h). During recovery following SD, $DA^{OxR2-KO}$ mice regain only ~40% as much REMS time compared to controls by end of the recovery dark phase (9.0±2.6 vs 23.8±4 min, *P*=0.007, independent *t*-test).
(b) Time-course of number of bouts of NREMS (left), or REMS (right) after SD, calculated as accumulated number of episodes of each state compared to baseline. n=9 mice per group, except n=10 for $DA^{OxR1\&2-CT}$.

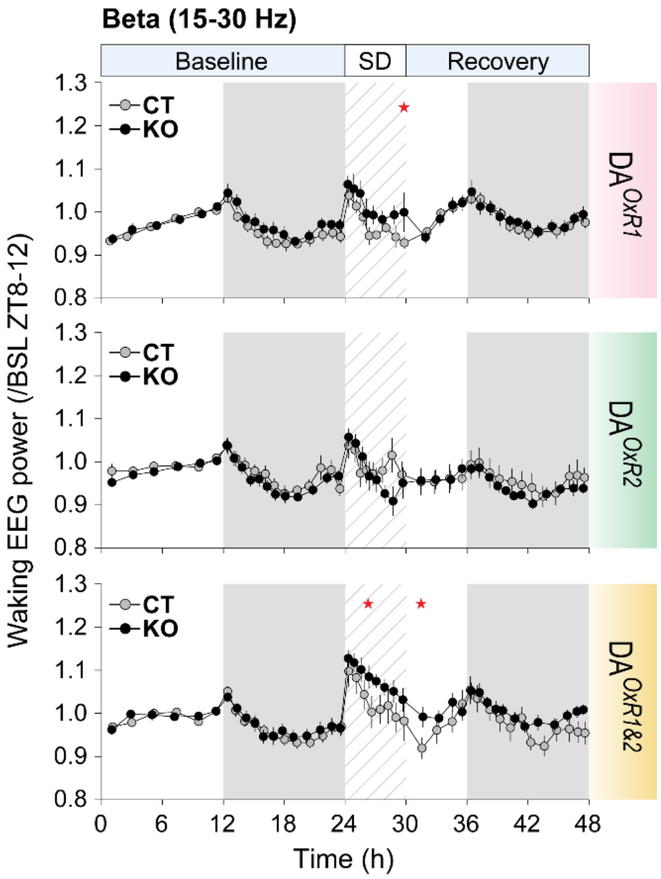

Figure S6: Upregulated EEG beta band activity in wakefulness of dopaminergic *Hcrtr1&2* doubly-ablated mice during sleep deprivation.

Dynamics of wakefulness beta band (15-30 Hz) power across baseline, SD and recovery. Beta power values are expressed relative to their average values during wakefulness of the last 4 h of baseline light phase. DA$^{OxR1\&2\text{-}KO}$ mice show enhanced beta during locomotion-associated enforced wakefulness compared to controls (two-way ANOVA; SD: genotype effect F(1,23)=23.300, *P*<0.001; with Bonferroni post-hoc test, **P*<0.05). n=9 mice per group, except n=10 for DA$^{OxR1\&2\text{-}CT}$.

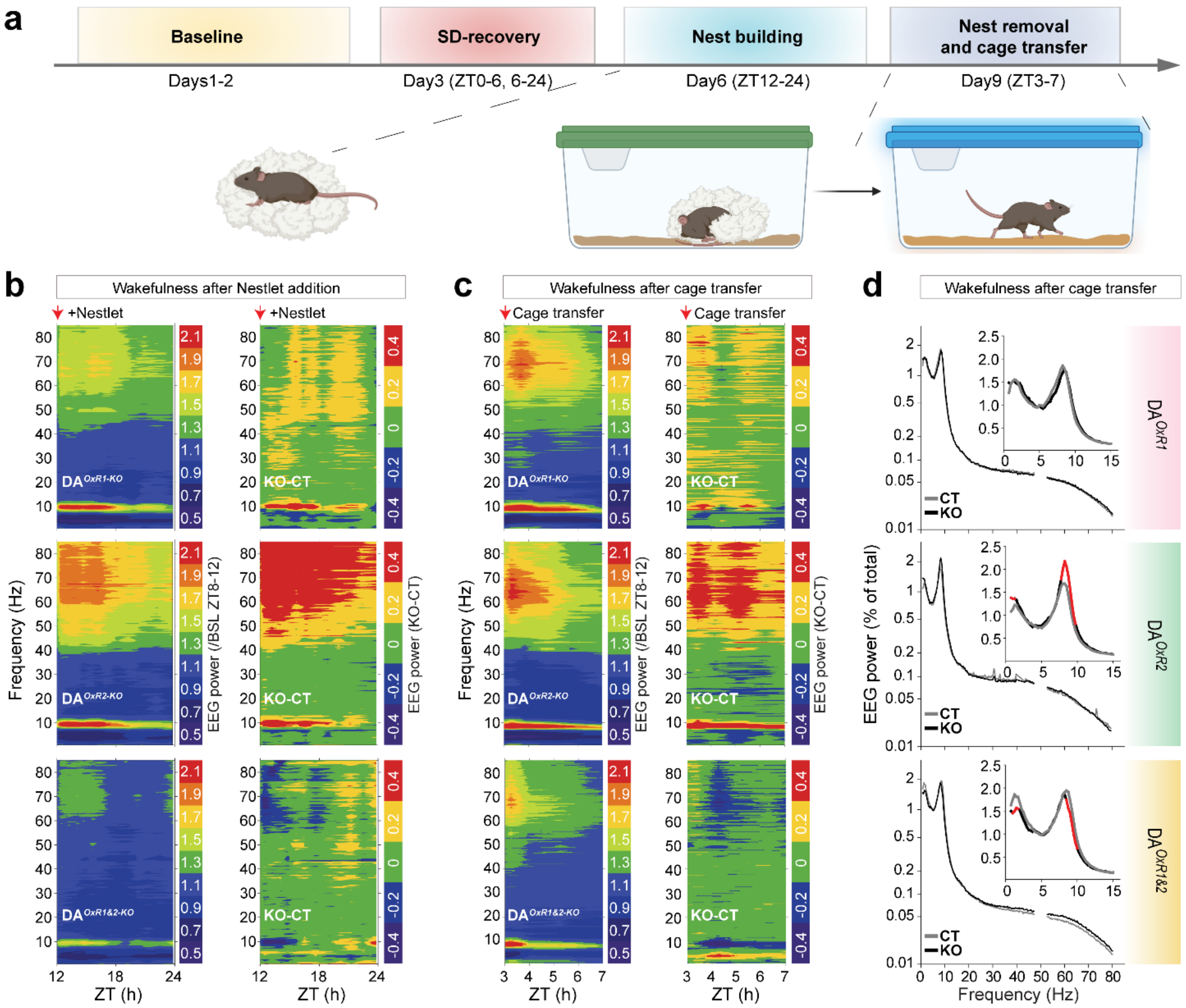

Figure S7: Dopaminergic *Hcrtr2*-ablated mice exhibit profound increases in waking theta and fast-gamma activity in both rewarding and stressful environments.

(**a**) Schematic of experimental design. Mice were provided with a preferred nesting material (a Nestlet^{TM}, see Methods) at dark-onset (ZT12) of Day 6 of a 9-day experimental timeline. On Day 9, mice were removed from the nest they had built and transferred to a fresh cage at ZT3, i.e., during an otherwise major sleeping period.

(**b,c**) Time-frequency heatmaps show the spectral dynamics of wakefulness from (b) the time when nesting material was added at dark-onset (ZT12) of Day-6 until the next light-onset (ZT24), and (c) from nest removal/cage transfer until the next sleep-onset. In the left heatmaps of both b and c, colors encode the average EEG power in each 0.25-Hz frequency bin, and across each time bin, expressed relative to their mean values in the last 4 h (ZT8-12) of baseline (BSL) light phase. Right heatmaps represent the differential dynamics of the waking EEG of KO and CT mice, where the power values of KO mice are subtracted from their controls (KO-CT).

(**d**) EEG PSD profile of wakefulness from nest removal and cage transfer to the next sleep-onset. Insets show magnification of spectra across 0.75-15 Hz. Red lines indicate significant differences. $DA^{OxR2\text{-}KO}$ mice exhibit higher theta power across 7.75-9.75 Hz, while $DA^{OxR1\&2\text{-}KO}$ mice show decreased power across 8.5-10 Hz, relative to controls (two-way ANOVA; $DA^{OxR2\text{-}KO}$: genotypeXfrequency interaction F(280,3653)=1.243, *P*=0.005; $DA^{OxR1\&2\text{-}KO}$: genotype effect F(1,286)=6.517, *P*=0.011; with Tukey post-hoc test, *P*<0.05). DA^{OxR1} (n=7:9 KO:CT), DA^{OxR2} (n=8:7 KO:CT), $DA^{OxR1\&2}$ (n=7:8 KO:CT).

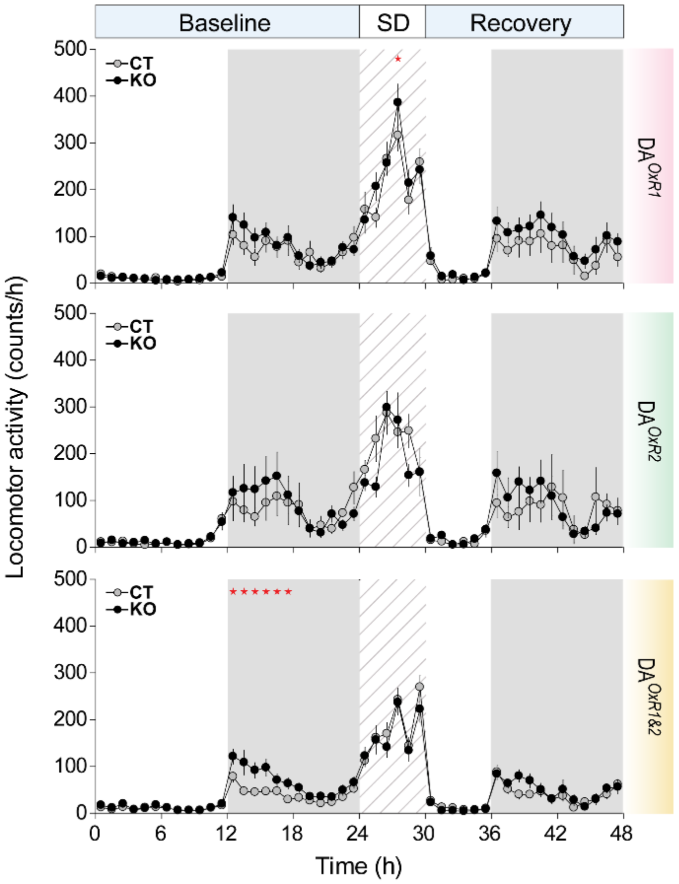

Figure S8: Locomotor activity in dopaminergic *Hcrtr1*, *Hcrtr2,* and double mutant mice.

The mice movements were monitored by infrared beam breaks, and locomotor activity (counts/h) is plotted across time during baseline (0-24 h, average of 2 baseline days), 6-h sleep deprivation (24-30h), and recovery (30-48h). The pronounced increase in theta dominated wakefulness of DA$^{OxR2\text{-}KO}$ mice is not coupled to increased locomotor activity. DA$^{OxR1\text{-}KO}$ mice showed higher activity during one timepoint in SD, but not during baseline or recovery. DA$^{OxR1\&2\text{-}KO}$ showed higher activity during the first half of baseline dark phase, but not during SD or recovery (two-way ANOVA; DA$^{OxR1\text{-}KO}$: SD: genotype effect $F(1,23)=6.792$, $P=0.009$; DA$^{OxR1\&2\text{-}KO}$: baseline: genotype effect $F(1,23)=38.381$, $P<0.001$, genotypeXtime interaction: $F(23,524)=2.646$, $P<0.001$; with Bonferroni post-hoc test, $*P<0.05$). n=9 mice per group, except n=10 for DA$^{OxR1\&2\text{-}CT}$.

Table S1: Electrophysiological features of putative histaminergic neurons of the tuberomammillary nucleus (TMN).

Passive membrane properties						
**Genotype**	**#**	**Cell capacity (pF)**	**τ membrane charging (ms)**	**τ membrane discharging (ms)**	**τ sag (ms)**	**Vrest (mV)**
C57BL/6J	23	21.00±0.71	17.50±1.34	20.97±1.78	979.8±96.4	-48.34±1.38
Hcrtr2-flox	18	21.44±1.16	20.57±2.61	20.55±1.96	855.2±126.4	-46.45±2.33
Hcrtr2-del	20	20.75±1.05	21.58±2.55	23.34±2.09	643.4±99.6	-51.80±1.88

Action potential features									
**Genotype**	**#**	**Amplitude (mV)**	**AHP (mV)**	**Half width (ms)**	**Rise slope (mV/ms)**	**Decay slope (mV/ms)**	**Threshold (mV)**	**Trigger slope (mV/ms)**	**$4^{th}/1^{st}$ spike interval**
C57BL/6J	23	72.3±2.4	-11.5±0.5	1.9±0.05	136.9±10.6	-37.9±1.2	-36.5±0.8	0.27±0.12	1.33±0.13
Hcrtr2-flox	16	67.3±2.6	-10.3±0.5	2.1±0.12	112.5±8.2	-34.3±2.0	-38.9±1.3	0.23±0.04	1.18±0.10
Hcrtr2-del	20	58.6±4.3	-11.2±0.6	2.4±0.18	88.3±13.4	-29.4±3.6	-31.4±3.8	0.14±0.04	1.28±0.13

Table S2: Comparison of vigilance states and EEG spectral profiles between conditional knockout mice and their respective controls.

Parameter	$DA^{OxR1\text{-}KO}$ *vs* $DA^{OxR1\text{-}CT}$	$DA^{OxR2\text{-}KO}$ *vs* $DA^{OxR2\text{-}CT}$	$DA^{OxR1\&2\text{-}KO}$ *vs* $DA^{OxR1\&2\text{-}CT}$
Wake baseline light	-	-	-
Wake baseline dark	-	-	Increased
NREMS baseline light	-	-	-
NREMS baseline dark	-	-	Decreased
REMS baseline light	-	Increased	-
REMS baseline dark	-	-	-
Wake recovery light	-	-	-
Wake recovery dark	-	Increased in 1st part	-
NREMS recovery light	-	-	-
NREMS recovery dark	-	Decreased in 1st part	-
REMS recovery light	-	-	-
REMS recovery dark	-	Decreased in 1st part	-
REMS state stability	-	increased	-
Wake EEG in baseline	-	Decreased in 2.75-5.0 Hz<br>Increased in 6.5-10 Hz	Decreased in 2.0-7.25 Hz<br>Increased in 8.5-9.75 Hz
REMS EEG in baseline	-	Increased 6.0-8.25 Hz	Decreased in 5.5-8.75 Hz
Wake EEG during sleep deprivation	Increased in 9-10.5 Hz	Decreased in 2-6 Hz<br>Increased in 7-10.75Hz	-
Wake EEG after cage change	-	Decreased in 0.75-1.25 Hz<br>Increased in 7.75-9.75 Hz	Decreased in 1-1.75 Hz and 8.5-10 Hz

Table S3: Comparison of waking theta peak frequency (TPF, Hz) between conditional knockout mice and their respective controls in different behavioral contexts. TDW, Theta-Dominated Wakefulness. CC, Cage Change. n=9 mice per group, except n=10 for $DA^{OxR1\&2\text{-}CT}$.

**Condition**	**$DA^{OxR1\text{-}KO}$ *vs* $DA^{OxR1\text{-}CT}$**	**$DA^{OxR2\text{-}KO}$ *vs* $DA^{OxR2\text{-}CT}$**	**$DA^{OxR1\&2\text{-}KO}$ *vs* $DA^{OxR1\&2\text{-}CT}$**
TDW in 48 h baseline	8.00±0.19 vs 7.91±0.12 Hz	8.08±0.16 vs 8.06±0.13 Hz	7.89±0.11 vs 7.7±0.10 Hz
Enforced wakefulness (SD)	8.47±0.18 vs 8.36±0.09 Hz	8.42±0.07 vs 8.11±0.17 Hz	8.22±0.16 vs 8.28±0.11 Hz
CC-induced wakefulness	8.18±0.25 vs 8.11±0.20 Hz	8.09±0.31 vs 8.11±0.13 Hz	8.21±0.06 vs 8.38±0.15 Hz

SI Text: Materials and Methods

Animals. Mice for analysis were produced by intercrossing *Hcrtr1 or* $2^{flox/flox}$ mice, with one parent heterozygous for $Dat^{IRES\text{-}Cre}$ (Slc6a3$^{tm1.1(cre)Bkmn}$). This cross generates two offspring groups: *Hcrtr1 or* $2^{flox/flox}$, $Dat^{+/IRES\text{-}Cre}$ (KO), and *Hcrtr1 or* $2^{flox/flox}$, $Dat^{+/+}$ (CT) mice. Note that each line has its own genetic control group. Mice are in a mixed *C57BL/6NTacXC57BL/6J* background. $Dat^{+/IRES\text{-}Cre}$ mice show Cre-mediated reporter expression at E17, suggesting *Hcrtr1* and *Hcrtr2* gene inactivation occurs in late gestation in our mice. To rule out effects of *Dat-IRES-Cre*, we recorded mice segregating solely the *Dat-IRES-Cre* allele ($Dat^{+/IRES\text{-}Cre}$ and $Dat^{+/+}$). None of the phenotypes we describe in DA^{OxR1}, DA^{OxR2} or $DA^{OxR1\&2}$-ablated mice were observed. In contrast, theta power of $Dat^{+/IRES\text{-}Cre}$ tended to be lower in wakefulness and REMS compared to $Dat^{+/+}$ mice. Mice were housed with food and water ad libitum under 12:12 light:dark cycle (lights-on at 08:00 AM, Zeitgeber Time ZT0). Animals were randomly distributed for sleep recording sessions and investigators performing SD and sleep scoring were blind to the animals' genotypes.

***Hcrtr2* gene targeting.** Two contiguous NcoI fragments (8.1 kb and 2.1 kb) from PAC clone RP23-392M3 of a *C57BL/6J* genomic library (BACPAC Resources Center, Oakland, CA, USA) containing *Hcrtr2* exon 1 and intron 1 were subcloned and used to construct the targeting vector. The targeting vector was designed to insert a 5'loxP site 70 bp downstream of the transcription start site (TSS) and 37 bp upstream of the ATG initiation codon within Exon 1. The 3'loxP site is inserted ~1 kb within Intron 1 followed by a promoter-less GFP coding sequence and a rabbit polyadenylation signal. This is designed so that, after Cre/loxP-recombination, the *Hcrtr2* gene promoter drives expression of *Gfp* instead of *Hcrtr2*, thus creating an allele acting both as KO and GFP-reporter. The targeting vector was linearized and electroporated into IC1 cells (*C57BL/6NTac* embryonic stem (ES) cell line, ingenious targeting laboratory, Ronkonkoma, NY, USA). Single colonies were screened by Southern blotting using probes 5′ and 3′ external to the targeting vector and a *Gfp* internal probe. Two clones demonstrating correct recombination events were injected into *BALB/cAnNHsd* blastocysts. The FRT-flanked neo cassette was excised by mating the resulting chimeras with *Tg*(*ACT-FLPe*)^{9205Dym} mice. The resulting neo-excised conditional KO allele, $Hcrtr2^{tm1.1Ava}$ (MGI-ID:5637402), is referred to as $Hcrtr2^{flox}$.

Immunohistofluorescence and confocal microcopy. Mice (~16-week-old of either sex) were deeply anesthetized with sodium pentobarbital (100 mg/kg, i.p.), and transcardially perfused with 4% paraformaldehyde (pH 7.4). Brains were quickly removed, post-fixed in the same fixative for 2 h at 4°C and immersed successively in 15% (1 h) and 30% sucrose (o/n) at 4°C, frozen and stored at −80°C until sectioning. For each mouse, 48X20 um-thick coronal sections from the midbrain (bregma -2.92 to -3.88 mm) were collected on SuperFrost-Plus glass slides, of which 6 sections (1 of 8) (−2.92, −3.08, −3.16, −3.28, −3.40, -3.52 mm from bregma) were used for cell counts. The midbrain

was sub-divided into VTA, PBP, PN and SNc according to Paxinos Atlas. For immunostaining, sections were blocked in 2% BSA, 5% normal donkey serum, 0.3% TritonX-100 in TBS (pH 7.5) for 30 min at rt, and antibodies were applied in 1% BSA, 0.2% TritonX-100 in TBS, and incubated o/n at 4°C. When double staining involved the HCRTR1 antibody, an additional antigen-retrieval step was applied using Sodium Citrate (pH 6.0) at 55ºC for 7 min before blocking and primary antibody incubation. Antibodies were anti-tyrosine hydroxylase (TH) from mouse (Incstar, Cat#22941 1:5000), anti-GFP from chicken (Aves Labs, Cat#1020; 1:500), anti-CRE from rabbit (Novagen, Cat#69050-3; 1:500), and anti-HCRTR1 from rabbit (Origene, Cat#TA328918; 1:100-1:500). Donkey IgG secondary antibodies coupled to Alexa-594 or -488 fluorophores (1:500) were incubated for 1 h at room temperature. Images were acquired on an inverted confocal laser-scanning microscope (Zeiss LSM710 with 405, 488, and 561-nm lasers) using a 40x oil objective (EC plan-Neofluar 40x/1.30 Oil DIC M 27). Minimal image processing used parameters identical for control and KO groups.

Surgery and EEG recordings. Surgical implantation and EEG recordings were as described[5, 6]. Briefly, after implantation of EEG/EMG electrodes, the mice were allowed one week for recovery and one week for habituation to the recording setup. EEG/EMG signals were acquired using EMBLA™ system at 200 Hz sampling rate and scored using the Somnologica-3™ software (Medcare). All EEG data were acquired in 10-15-week-old males (27–31 g), housed individually after electrode implantation. Locomotor activity was monitored using infrared sensors and ClockLab software. Animals were exposed sequentially to four behavioral contexts (Baseline-SD-Nest-Cage change[7]. After two baseline recording days, a 6-h sleep deprivation (SD) was initiated at light-onset (ZT0) on day 3. Following SD, mice were left undisturbed for 2 days. At dark-onset (ZT12) of day 6, a square of packed shreddable cotton (Nestlet™, Ancare, Bellmore, NY, CatNr. 14010) was introduced into the cage. Mice were left undisturbed until next light-onset with assessment of nest morphology. Mice were left undisturbed for another 2 days. On ZT3 of day 9, mice were transferred from their home cage where the nest had been built to a fresh cage. Latency to NREMS-onset was defined as the time until the first ≥2 min-long NREMS episode. Animals showing epileptic-like seizures or pre-epileptic-like spikes were excluded from analysis (~10% of mice).

Vigilance state analysis. We scored vigilance states manually, blind to the experimental conditions, in 4-s epochs by concurrent evaluation of EEG and EMG signals in the Somnologica-3™ software (Medcare) using established criteria[6]. Scripts were developed to quantify wakefulness, NREMS, REMS episode number, duration and vigilance state fragmentation as previously described[5, 6, 8]. TDW analysis was performed as described in Vassalli and Franken[6]. REMS latency was calculated as time from sleep-onset (first NREMS episode ≥12 s) to the first REMS episode following SD.

EEG power spectral density and time-course analysis. EEG signals were subjected to discrete Fourier transform with non-overlapping 4-s hamming windows to determine power spectral densities

(PSD) across 0.25-80 Hz with 0.25 Hz frequency-bin resolution[5, 6]. For each vigilance state and time interval, PSDs of all artifact-free, same-state-flanked 4-s epochs were averaged to generate mean PSDs for each animal. To account for differences among animals in absolute EEG power, power density in each frequency bin and for each state was expressed as percentage of a baseline reference value, calculated for each mouse across 2 baseline days by summation of the power across 0.75-40 Hz frequency bins in all 3 behavioral states. This reference value was weighted so that for each animal each state contributed equally to the total EEG power[9]. To analyze how specific spectral components of the waking EEG evolve across time, EEG power density within delta (1-4 Hz), inter-delta/theta (4-7 Hz), theta (7-11 Hz), beta (15-30 Hz) and fast-gamma (52-80 Hz) bands were first normalized by the mean power density within that frequency range during wakefulness of the last 4 h of the two baseline light phase (ZT8-12; time of minimal sleep pressure), then averaged across each time interval, and each individual mouse of each genotype, and plotted across time. The number of time intervals were adjusted according to the prevalence of wakefulness, i.e., 6 during baseline light phase, 12 in dark phase, 8 during the 6-h SD, and 4 in recovery light phase. To generate time-frequency heatmaps of EEG power during wakefulness, power density in each 0.25 Hz frequency bin was as above first expressed relative to its average value during wakefulness of baseline light phase ZT8–12, and then averaged across each time interval, and individual mouse of each genotype. Time intervals are the same as for time-course analyses of the power in each frequency band, as indicated above.

Detection of phasic REMS events. We detected phasic REMS events, which are transient increase in theta power and frequency during REMS, as described previously[10,11]. Briefly, we first bandpass-filtered EEG signal between 4 and 12 Hz using finite impulse response filters with an order equal to three cycles of the low cutoff frequency, and detected the individual theta peaks from the filtered signal. We then smoothed the interpeak interval time-series using an 11-sample moving average window, and selected the smoothed interpeak intervals shorter than the 10th percentile as candidate phasic REMS events. The candidate events with the following criteria were considered as phasic REMS: (1) minimum event duration of 900 ms; (2) minimum smoothed interpeak interval shorter than 5th percentile; (3) mean amplitude of theta peaks larger than mean amplitude of theta peaks across all REMS.

Theta-gamma cross-frequency coupling. We used the modulation index (MI) to measure theta-gamma phase-amplitude coupling[12, 13]. Using finite impulse response filters with an order equal to three cycles of the low-cutoff frequency, we bandpass-filtered EEG signals into theta (7-11 Hz) and fast-gamma (52-80 Hz) in both forward and reverse directions to eliminate phase distortion. We then estimated instantaneous phase of theta and the envelope of fast-gamma using the Hilbert transform. Theta phase was discretized into 18 equal bins (N=18, each 20°) and the average value of fast-gamma envelope within each bin was calculated. The resulting phase-amplitude histogram (P) was

compared with a uniform distribution (*U*) using the Kullback-Leibler distance, $D_{KL}(P,U) = \sum_{j=1}^{N} P(j) * log[P(j)/U(j)]$, and normalized by *log(N)* to obtain the modulation index, $MI = D_{KL} / log(N)$. For each animal, we filtered continuous 48 h baseline recordings into the theta and fast-gamma bands and estimated the Hilbert transform, and then concatenated episodes of each state to calculate the MI during dark and light phases. To explore possible coupling patterns between different pairs of low and high-frequency bands, we used comodulogram analysis[12]. We considered 16 frequency bands for phase (1-18 Hz, 1-Hz increments, 2-Hz bandwidth), and 14 frequency bands for amplitude (15-90 Hz, 5-Hz increments, 10-Hz bandwidth). MI values were then calculated for all these pairs to obtain the comodulogram graph.

Three-choice serial reaction time task (3-CSRTT). Adult males and control littermates (5-7 months-old) were group-housed (3-5 animals/cage) under a 12-h reversed light/dark cycle (light-onset at 8:30 p.m.) at a constant temperature (22°C) and had *ad libitum* access to water. The 3-CSRTT was conducted in mouse operant chambers (15cmx15cmx13.5 cm, Med Associates, St Albans, VT, USA), each enclosed in a wooden cubicle. Chambers featured an exhaust fan for ventilation, serving as white noise emitter for sound attenuation, a steel grid floor, three 20-mm horizontally-spaced nosepoking holes on one wall, and a reward receptacle with a liquid dipper and a 0.01-ml-cup on the opposite wall. Lights could be illuminated inside each nosepoking port, the reward receptacle, and on the chamber ceiling. Mice were trained to self-administer a 0.2% saccharine liquid reward (Sigma-Aldrich). A nosepoke in the “active” port illuminated the aperture and activated delivery of a 0.01 ml reward, which remained available for 3 s once head entry in the liquid dipper was detected. Supplementary entries into the active port in the absence of head entry above the liquid dipper and entries in inactive ports were recorded but had no consequence.
The 3-CSRTT comprised four training stages and a test phase: Stage 1: Mice learned to perform a head entry into the reward magazine to receive 0.01 ml saccharine. Mice were considered to have reached training criteria once they reached 50 rewards in 30 min. Stage 2: When the mouse head entered the reward magazine, a cue light stimulus appeared randomly in one of the three nosepoking ports and stayed lit until a nosepoke in this hole occurred, upon which the light turned off, the dipper was activated and the reward made available (even if the mouse additionally nosepoked in any of the two other unlit apertures). Three seconds after the reward was made available, the dipper deactivated, and a light cue appeared once again randomly in one of the three ports. This went on until the mouse earned 40 rewards during a 30-min training. Stage 3: similar conditions as “Stage 2”, except that a 5-s delay separated beginning of a trial by magazine head entry and illumination of one of the ports. A correct response is a nosepoke in the illuminated port, which triggers a reward, and an incorrect response is a nosepoke in any other unlit port, which triggers illumination of the ceiling light for 5 s and no reward. After each correct or incorrect response, a new trial begins with head entry into the reward magazine. This went on until mice earned 50 rewards during the 30-min training. Stage 4: after trial start by nosepoking in the reward magazine, a 5-s-delay, like in Stage 3,

preceded illumination of one of the ports, but the cue light stayed illuminated for only 2 s. Any response before cue light illumination is a premature response, without programmatic consequences. Omissions were recorded when no nosepoking occurred during the allowed 5 s after beginning of illumination of one the apertures. To advance to the "Test phase", the mice had to earn ≥30 rewards during the 30-min session. Mice underwent 2 sessions per day. Usually one day, in some cases two days, were necessary to reach next-stage criteria. The Test phase was as "Stage 4" except that premature responses during the 5-s delay were followed by a 5-s time-out period with illumination of the ceiling light. A perseverative response was defined as repetitive nosepoke in the liquid dispenser after reward consumption and served as measure of compulsive behavior. Premature, correct, incorrect, omission, and perseverative responses were recorded. Sessions of the 3 last days in "Test phase" were averaged for each mouse to establish baseline performance.

Attention and motivation probe. The conditions in the attention probe were the same as during the "Test phase", except that the cue light duration was 3 s (easier than in the former test phase), then 2 s (as in test phase) and finally 1 s, making the task contingencies progressively more difficult. Nosepoking before cue light illumination had no consequence, hence no premature response was recorded. Only a correct response gives access to the reward, nosepoking in the 2 unlit apertures triggers an incorrect response and no reward. During the motivation probe sessions, the mice' motivation to poke for rewards was tested without the attentional constraints above. One and the same hole was active throughout the test. Mice were first tested under a fixed ratio of 1, where one nosepoke in the active port provides one reward. The next day mice were submitted to a progressive-ratio schedule of reinforcement, which consisted of a systematic within-session increase in number of responses required to earn one reward. The number of active nosepokes required increased from reward to the next according to the progression sequence: response ratio (rounded to nearest integer) = $(5e^{reward \times 0.2})-5$[14]. Hence, the progressive-ratio schedule followed: 1, 2, 4, 6, 9, 12, 15, 20, 25, 32, 40, 50, 62, 77, 95, 118, 145,178, 219, etc.

Patch-clamp whole-cell recording from TMN brain slices. A total of 16 young adult *C57BL/6J* (Charles River, USA), 13 *Hcrtr2*$^{flox/flox}$, and 17 *Hcrtr2*$^{del/del}$ mice of both sexes (7m/9f, 6m/7f, and 9m/8f, respectively), aged P21-P49, were maintained in pathogen-free conditions, with free access to food and water, and a 12:12 light:dark cycle. Procedures followed Italian law (2014/26, implementing 2010/63/UE) and were approved by the local Ethical Committee and the Italian Ministry of Health. After deep anesthesia with 5% isoflurane, mice were decapitated and brains rapidly extracted and placed in ice-cold solution, containing (mM): 87 NaCl, 21 $NaHCO_3$, 1.25 NaH_2PO_4, 7 $MgCl_2$, 0.5 $CaCl_2$, 2.5 KCl, 25 D-glucose, 75 sucrose, 0.8 ascorbic acid, aerated with 95% O_2 and 5% CO_2 (pH 7.4). Coronal slices (300 µm-thick) from the TMN were cut between -2.18 and -2.92 mm from bregma, with a VT1000S vibratome (Leica Microsystems) and maintained at 30°C in the above solution for ≥1 h before transfer to the recording chamber. Cells were examined with an Eclipse

E600FN microscope (Nikon). Putative histamine neurons were identified using their reported electrophysiological properties[15,16]. Stimulation and recording were performed at 32-34°C with Multiclamp 700A (Molecular Devices). Micropipettes (2-4 MΩ) were pulled from borosilicate capillaries (Science Products GmbH) with a P-97 Flaming/Brown Micropipette Puller (Sutter Instruments). Cell capacitance and series resistance (generally <10 MΩ) were always compensated (up to 75%). Input resistance was usually 30-70 MΩ. Slices were perfused at 1.8-2 ml/min with artificial cerebrospinal fluid (ACSF) containing (mM): 129 NaCl, 21 $NaHCO_3$, 1.6 $CaCl_2$, 3 KCl, 1.25 NaH_2PO_4, 1.8 $MgSO_4$, 10 D-glucose, aerated with 95% O_2 and 5% CO_2 (pH 7.4). Pipettes contained (mM): 140 K-gluconate, 5 KCl, 1 $MgCl_2$, 0.1 BAPTA, 2 Mg ATP, 0.3 Na-GTP, 10 HEPES (pH 7.25). In some experiments, 0.15% biocytin was added for post-recording staining and morphological reconstruction. Traces were low-pass Bessel filtered at 2 kHz and digitized at 10 kHz, with pClamp9/Digidata 1322A (Molecular Devices). The resting membrane potential (V_{rest}) was measured in open circuit mode, soon after obtaining the whole-cell configuration. No correction was applied for liquid junction potentials.
Orexin B (OXB), [Ala11,D-Leu15]-Orexin B (OXB-Ala,Leu), and (2S)-1- (3,4-dihydro-6,7-dimethoxy-2(1H)-isoquinolinyl)-3,3-dimethyl-2-[(4-pyridinylmethyl)amino]-1-butanone hydrochloride (TCS-OX2-29) were from Tocris Bioscience (Bristol, UK), and dissolved in distilled water-based stock solutions, aliquoted and stored at -20°C until usage.

Analysis of patch-clamp data. Passive membrane properties, action potential (AP) features and firing responses to agonists were analyzed off-line using Clampfit 9.2 (Molecular Devices) and OriginPro 2019 (OriginLab Corporation, Northampton, MA, USA). All AP features were analyzed during the first step of current injection able to trigger multiple APs; individual AP parameters were measured on the first AP of the induced train. Spike width was calculated at half-amplitude, spike amplitude was computed as the difference between AP threshold and peak. Adaptation was measured as the ratio between the fourth and the first spike interval. Spike intervals were measured between consecutive peaks. After-hyperpolarization was the difference between the AP threshold and the most negative membrane potential (V_m) reached on repolarization. The triggering depolarization slope was the difference between the most negative V_m reached on repolarization and the following AP threshold, divided by the relative time. The time constants of membrane charging and discharging were estimated by mono-exponential fit to the passive response to -100 pA injection. For the I_h-related sag, τ was derived from mono-exponential fit to the current decay from the negative peak to the end of the 0.5 s current pulse (-50 pA). Drugs were perfused in the bath and their effects on cell firing were measured for 2 min after reaching the maximal effect, which in the case of HCRTR2 agonists usually occurred ~30 s after the administration ended. Only one neuron was sampled in each slice, to avoid uncontrolled long-term effects of neuromodulators (e.g. on receptor desensitization).

Putative histaminergic neurons were identified based on their typical electrophysiological properties, such as slow depolarization induced by step current injection, ~2 Hz spontaneous firing, late-spiking profile, I_h-related sag upon hyperpolarizing pulses, V_{rest} ~-50 mV and cell capacitance ~20 pF. The passive membrane properties, including τ's of membrane charging, discharging and I_h-related sag, as well as the main AP features were consistent across genotypes and with those previously reported[1].

Statistics. Animals of all genotypes were randomly distributed for sleep recording or behavioral sessions, and investigators involved in sleep scoring or data acquisition were blinded as to the animals' genotype. Statistical analyses were performed using SigmaPlot12.0 or GraphPad prism8.4.2. Data normality and variance homogeneity were respectively verified using the Shapiro-Wilk and the F test as well as by Q-Q plot inspection. Statistical significance of comparisons was determined using independent *t*-test, or two-way ANOVA, with *P*, *F*, *t* and *df* values reported in Figure legends. Significant ANOVA analyses were followed by Bonferroni or Tukey multiple comparison post-hoc tests. Values in the text are reported as mean ± standard error mean (SEM) unless reported otherwise. The number of experiments refers to the number of animals, or neurons of different brain slices.

Illustration software. Fig. 1b, Fig. 2a, Fig. S1b and Fig.S7a were created with the help of BioRender.com. All Figures were prepared using Adobe Illustrator CC (Adobe).

SI References

1. Haas HL, Reiner PB. Membrane properties of histaminergic tuberomammillary neurones of the rat hypothalamus in vitro. *J Physiol* 1988; **399:** 633-646.

2. Zhou FM, Hablitz JJ. Postnatal development of membrane properties of layer I neurons in rat neocortex. *The Journal of neuroscience : the official journal of the Society for Neuroscience* 1996; **16**(3)**:** 1131-1139.

3. Spigelman I, Zhang L, Carlen PL. Patch-clamp study of postnatal development of CA1 neurons in rat hippocampal slices: membrane excitability and K+ currents. *Journal of neurophysiology* 1992; **68**(1)**:** 55-69.

4. Sanchez-Aguilera A, Monedero G, Colino A, Vicente-Torres MA. Development of Action Potential Waveform in Hippocampal CA1 Pyramidal Neurons. *Neuroscience* 2020; **442:** 151-167.

5. Vassalli A, Dellepiane JM, Emmenegger Y, Jimenez S, Vandi S, Plazzi G *et al.* Electroencephalogram paroxysmal theta characterizes cataplexy in mice and children. *Brain* 2013; **136**(Pt 5)**:** 1592-1608.

6. Vassalli A, Franken P. Hypocretin (orexin) is critical in sustaining theta/gamma-rich waking behaviors that drive sleep need. *Proc Natl Acad Sci U S A* 2017; **114**(27)**:** E5464-E5473.

7. Li S, Franken P, Vassalli A. Bidirectional and context-dependent changes in theta and gamma oscillatory brain activity in noradrenergic cell-specific Hypocretin/Orexin receptor 1-KO mice. *Scientific reports* 2018; **8**(1)**:** 15474.

8. Bandarabadi M, Herrera CG, Gent TC, Bassetti C, Schindler K, Adamantidis AR. A role for spindles in the onset of rapid eye movement sleep. *Nat Commun* 2020; **11**(1)**:** 5247.

9. Franken P, Malafosse A, Tafti M. Genetic variation in EEG activity during sleep in inbred mice. *The American journal of physiology* 1998; **275**(4 Pt 2)**:** R1127-1137.

10. Mizuseki K, Diba K, Pastalkova E, Buzsaki G. Hippocampal CA1 pyramidal cells form functionally distinct sublayers. *Nature neuroscience* 2011; **14**(9)**:** 1174-U1235.

11. Hammer M, Schwale C, Brankack J, Draguhn A, Tort ABL. Theta-gamma coupling during REM sleep depends on breathing rate. *Sleep* 2021; **44**(12).

12. Tort AB, Kramer MA, Thorn C, Gibson DJ, Kubota Y, Graybiel AM *et al.* Dynamic cross-frequency couplings of local field potential oscillations in rat striatum and hippocampus during performance of a T-maze task. *Proc Natl Acad Sci U S A* 2008; **105**(51)**:** 20517-20522.

13. Bandarabadi M, Boyce R, Gutierrez Herrera C, Bassetti CL, Williams S, Schindler K *et al.* Dynamic modulation of theta-gamma coupling during rapid eye movement sleep. *Sleep* 2019; **42**(12)**:** 1-11.

14. Richardson NR, Roberts DC. Progressive ratio schedules in drug self-administration studies in rats: a method to evaluate reinforcing efficacy. *J Neurosci Methods* 1996; **66**(1)**:** 1-11.

15. Eriksson KS, Sergeeva O, Brown RE, Haas HL. Orexin/hypocretin excites the histaminergic neurons of the tuberomammillary nucleus. *The Journal of neuroscience : the official journal of the Society for Neuroscience* 2001; **21**(23)**:** 9273-9279.

16. Michael NJ, Zigman JM, Williams KW, Elmquist JK. Electrophysiological Properties of Genetically Identified Histaminergic Neurons. *Neuroscience* 2020; **444:** 183-195.
